# Integrated multi-omics reveals plasma biomarkers of fatigue in HIV immunological non-responders

**DOI:** 10.3389/fcimb.2026.1932594

**Published:** 2026-10-15

**Authors:** Baixue Li, Xi Chen, Jibin Liu, Li Wen, Zhengnan Shen, Hang Zhou, Peijie Wu, Quansheng Feng

**Affiliations:** 1 School of Basic Medicine, Chengdu University of Traditional Chinese Medicine, Chengdu, China; 2 Traditional Chinese Medicine Department, Jianyang Maternal and Child Health Hospital, Jianyang, China

**Keywords:** aids, cytokine, fatigue, HIV, immune reconstitution deficiency, plasma lipids

## Abstract

**Objective:**

To characterize the metabolic and inflammatory signatures distinguishing fatigued from non-fatigued HIV immunological non-responders (INRs) and to explore candidate biomarkers and potential mechanistic pathways underlying HIV-associated fatigue.

**Methods:**

We performed untargeted metabolomics and cytokinomics on plasma obtained from 18 fatigued and 17 non-fatigued HIV-INRs. Mechanistic insights were explored via Protein-Protein Interaction (PPI) networks and Kyoto Encyclopedia of Genes and Genomes (KEGG) enrichment analyses.

**Results:**

Compared to non-fatigued controls, the fatigued cohort showed significant up-regulation of 4-ketopimelic acid, cholesterol, and 1-hexadecyl-sn-glycero-3-phosphocholine, with concurrent down-regulation of DL-arginine and D-pipecolic acid (P < 0.05). Immunologically, the fatigued group displayed elevated macrophage migration inhibitory factor (MIF) but reduced interferon-induced protein 10 (IP-10/CXCL10) and interleukin-18 (IL-18) levels (P < 0.05). Functional enrichment analysis suggested the involvement of necroptosis, lipid metabolism, and atherosclerosis signaling pathways.

**Conclusion:**

Fatigue in HIV-INRs receiving long-term antiretroviral therapy was associated with distinct lipidomic and cytokinomic alterations. These results underscore the potential roles of dysregulated fatty acid metabolism, oxidative stress, and chronic low-grade inflammation in the pathophysiology of fatigue. Cholesterol and IL-18 emerged as candidate biomarkers associated with HIV-associated fatigue; however, their combined discriminatory performance was not statistically validated in this exploratory study and requires confirmation in larger, independent cohorts.

## 1 Introduction

Antiretroviral therapy (ART) enables most people living with Human Immunodeficiency Virus (HIV)/Acquired Immunodeficiency Syndrome (AIDS) (PLHIV) to achieve viral suppression, typically with a significant increase in Cluster of Differentiation 4+ T-Cell (CD4+ T-cell) counts. However, in approximately 15%–30% of PLWH, particularly those with prolonged infection before treatment, the resident CD4+ T-cell response fails to recover fully despite sustained viral suppression, a condition known as incomplete immune reconstitution. According to the US Department of Health and Human Services, people living with HIV (PLWH) who fail to achieve CD4+ T-cell counts above 350–500 cells/mL following 4–7 years of suppressive antiretroviral therapy are classified as immunological non-responders (INRs) (Gazzola et al., 2009; Yang et al., 2020). Moreover, INRs have a significantly higher risk of opportunistic infections, long-term complications, and all-cause mortality, rendering incomplete immune reconstitution a critical focus of post-ART studies.

Many PLHIV experience various comorbidities and related conditions, with fatigue being one of the most common and debilitating conditions. International studies have indicated that the prevalence of fatigue ranges from 33% to 88% among PLHIV (Henderson et al., 2005; Jong et al., 2010), compared with 7% to 42% in the general population (Finsterer and Mahjoub, 2014). Fatigue is characterized by an intense sensation of tiredness, low energy, inertia, or weakness that worsens with activity and impairs normal functioning. In PLHIV, fatigue may contribute to depression, reduce medication adherence, increase drug resistance, and impair physical, psychological, and social functioning, thereby diminishing the overall quality of life (Langseth et al., 2022). Consequently, assessing fatigue is essential for evaluating the quality of life of patients. An analysis of fatigue status and its influencing factors among PLHIV revealed that age, CD4+ T-cell count, and sleep quality are the primary factors affecting overall fatigue levels (Saito et al., 2024). Although the prevalence of fatigue in INRs is rarely documented in the literature, the established association between fatigue and immune function, along with our clinical observations, suggests that fatigue is likely a common symptom in INRs and warrants further investigation.

The pathophysiological mechanisms underlying fatigue in PLHIV have been linked to mitochondrial dysfunction, chronic inflammation, and neuronal pathology (Thangaraj et al., 2018; Nass et al., 2023; Elahi et al., 2024). However, in most cases, a definitive cause cannot be determined. Although numerous studies have indicated that fatigue levels are strongly associated with CD4+ T-cell count or immune status, conflicting reports exist on the association between fatigue and disease progression, CD4+ T-cell count, or HIV viral load (VL) (Sullivan et al., 2003; Barroso et al., 2010), at least partially due to the subjective assessment of fatigue. Therefore, identifying objective fatigue biomarkers is clinically and socially significant.

In this context, metabolic profiling has become a promising noninvasive method for diagnosing and monitoring HIV-infected patients with varying immunological responses to long-term ART. Numerous studies have reported significant alterations in lipid and amino acid metabolite levels in INRs and Immunological Responders (IRs). Furthermore, lipid metabolism and signaling are believed to be essential in inflammation, underscoring the potential of lipidomic profiling for diagnosing INRs. However, the relationship between fatigue and changes in the plasma lipidome in INRs remains unexplored.

To address this gap, we aimed to thoroughly evaluate the immune-metabolic adaptations in INRs with and without fatigue to identify a metabolic phenotype associated with fatigue. Using ultra-performance liquid chromatography-time-of-flight mass spectrometry, we performed unsupervised and supervised analyses to investigate the statistically significant differences in metabolic profiles associated with fatigue in INRs. Collectively, these findings hold promise for guiding novel therapeutic interventions to improve the health status of immunological non-responders (INRs) living with HIV.

## 2 Materials and methods

### 2.1 Ethical considerations

This study was approved by the Ethics Committee of the Affiliated Hospital of Chengdu University of Traditional Chinese Medicine (No. 2018KL-062). All procedures were conducted in accordance with relevant guidelines and regulations and in compliance with the principles of the Declaration of Helsinki. Written informed consent was obtained from patients with INRs before sample collection and analysis of demographics and clinical data.

### 2.2 Study participants

Participants who met the inclusion criteria were recruited between January 2022 and December 2023 from the Zhaojue County Center for Disease Control and Prevention and the Butuo County Center for Disease Control and Prevention in Liangshan Prefecture, Sichuan Province, China. All participants had completed at least 3 years of continuous clinical monitoring and had maintained suppressed VL (< 50 copies/mL). INRs were defined as individuals who had undergone ART for over three years, achieved virological suppression, and exhibited CD4+ T-cell counts ≤ 350 cells/μL (**Figure 1**). The INR definition was based on CD4+ T-cell counts that remained ≤ 350 cells/μL throughout long-term follow-up under virological suppression; the CD4+ T-cell counts shown in **Figure 2F** are single values measured at the sampling visit, and a transient rebound above 350 cells/μL at sampling did not alter a participant’s INR classification. CD4+ T-cell counts at ART initiation, at the nadir, and at enrollment are reported for each participant in **Supplementary Material 2**. Among the participants, 35 INRs completed fatigue assessments, including the Fatigue Scale (FS) and psychological health-related quality-of-life measures. This group consisted of 18 fatigued and 17 non-fatigued INRs. The two INRs subgroups were matched for baseline CD4+ T-cell count, VL, age, and sex. Individuals co-infected with the hepatitis B or hepatitis C virus were excluded. Furthermore, 10 HIV-seronegative healthy controls (HCs) matched for age and sex with the INRs were recruited as the control group (**Figure 1**). Immunophenotyping (CD4+ and CD8+ T-cell counts and the CD4+/CD8+ ratio) was performed at the same time point as plasma sampling, whereas the baseline matching described above was anchored to characteristics at enrollment.

**Figure 1:**
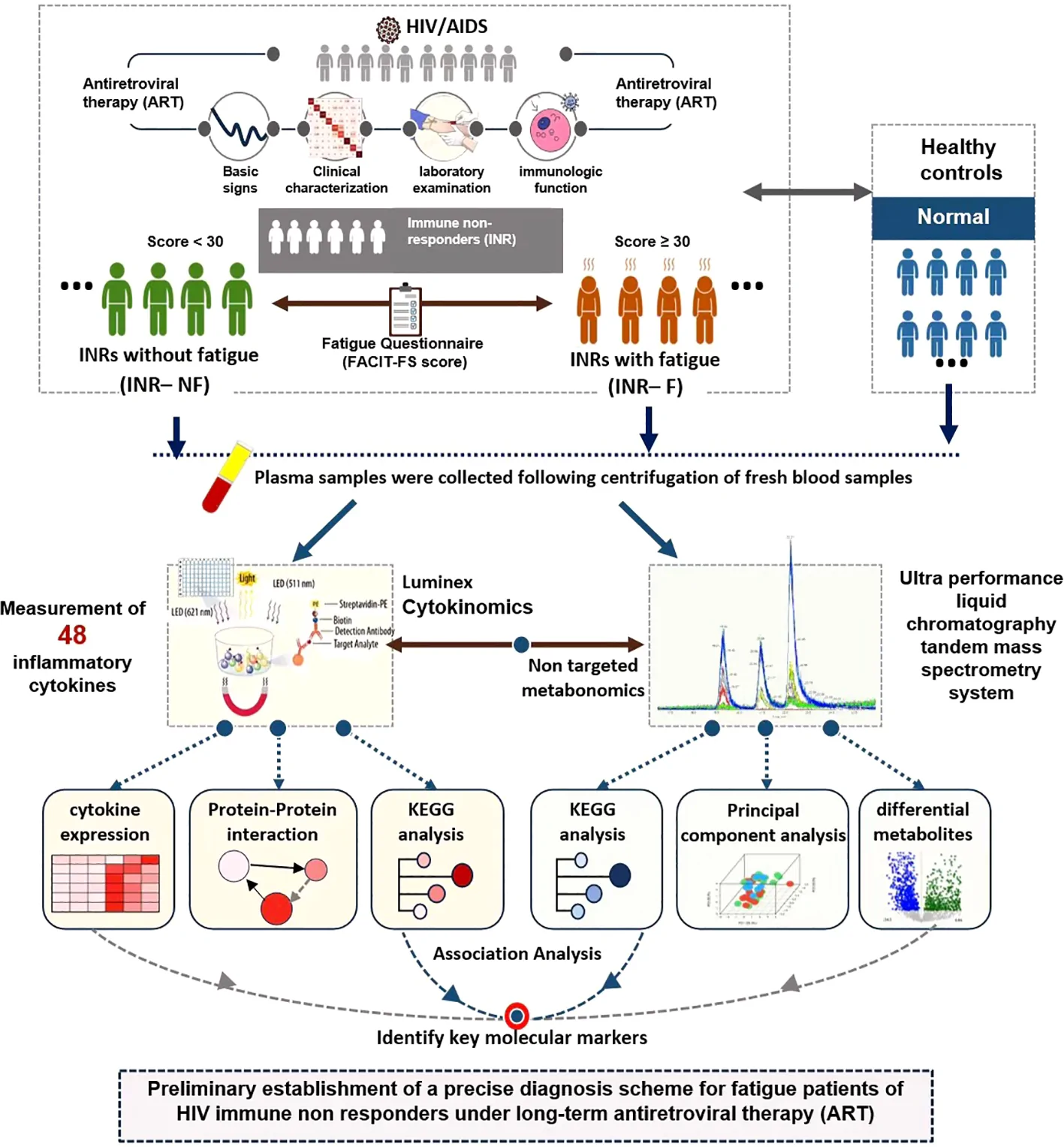
Schematic of the experimental design. INRs: Patients who have received ART for over 3 years, have achieved virological suppression (VL < 50 copies/mL), and exhibit CD4+ T-cell counts that remain ≤ 350 cells/μL during long-term follow-up, consistent with the definition in the Methods.

**Figure 2:**
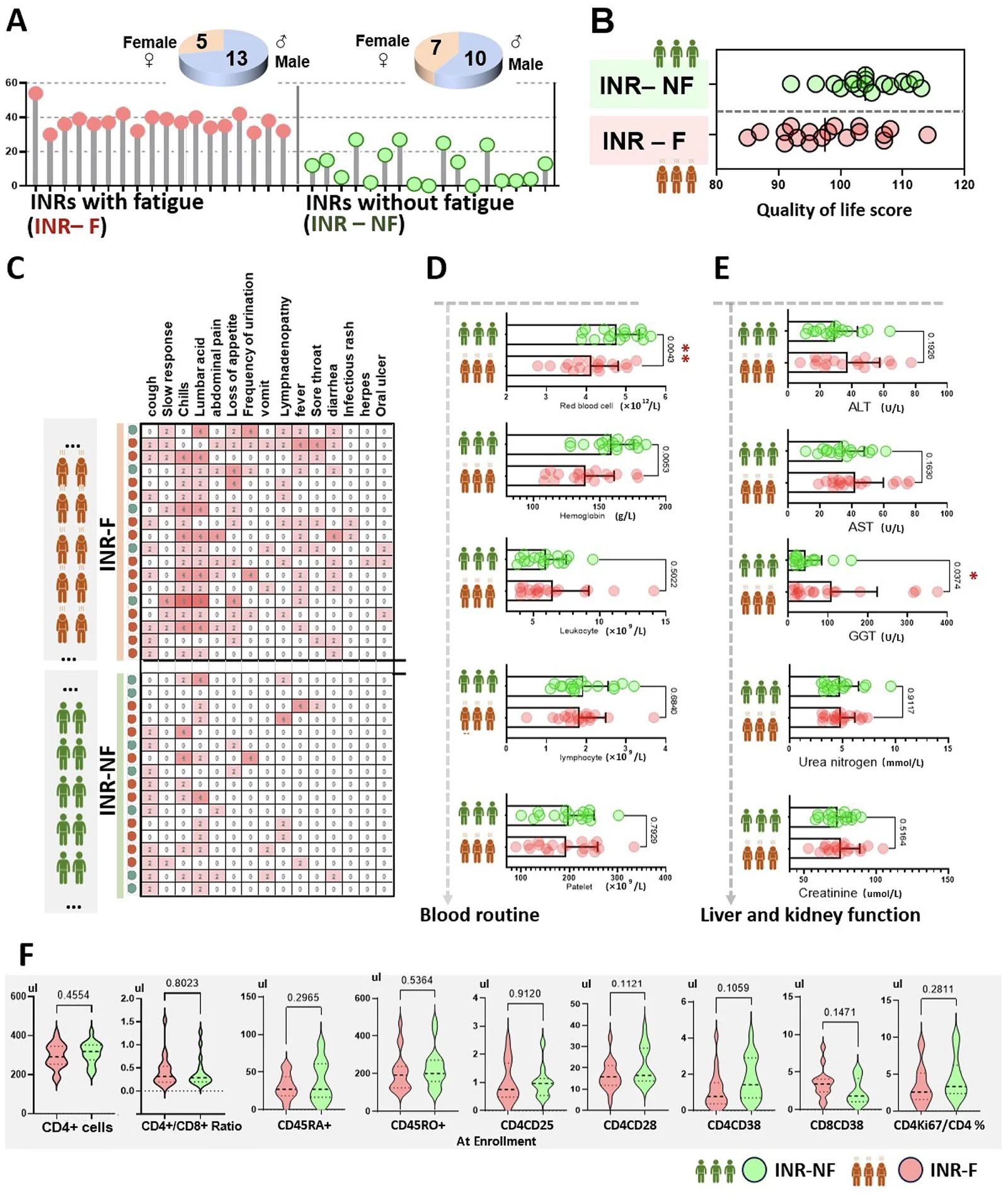
**(A)** Group-wise fatigue scores (modified FACIT-FS) and gender distribution. **(B)** Comparative analysis of MOS-HIV scale scores across groups. **(C)** Symptomomics-based differential analysis across groups. **(D)** Comparative analysis of hematological parameters among groups. **(E)** Comparative analysis of key hepatic and renal function indicators among groups. **(F)** Comparative analysis of T-cell immune parameters among groups.

### 2.3 Fatigue questionnaire (FACIT-FS score)

Fatigue was evaluated using the Functional Assessment of Chronic Illness Therapy-Fatigue Scale (FACIT-FS) questionnaire. A modified version of the FACIT-FS was developed following preliminary clinical evaluations in our outpatient setting. The modified scale comprises 15 items, each with five possible responses ranging from 0 (not at all fatigued) to 4 (very fatigued). The overall score for each subscale was the sum of the item scores, resulting in a potential range of 0 to 60, with higher scores indicating greater fatigue severity.

In this study, the modified FACIT-FS was used to assess fatigue severity among HIV-infected INRs. Based on previous research (Kiss et al., 2025; Regueiro et al., 2025; Tanaka et al., 2025), recommendations from the original scale developers, and our clinical observations, we observed that patients scoring above 30 exhibited more severe fatigue symptoms—such as mild cognitive impairment, reduced social activity, or other functional limitations—with a significant effect on daily living, whereas those scoring below 30 had milder symptoms. Therefore, a score of 30 was established as the threshold to distinguish between the fatigued and non-fatigued groups (**Supplementary Table S1**-**1** for details of the scoring scale).

### 2.4 Clinical characterization data collection

Medical Outcomes Study (MOS)-HIV Scale: The MOS-HIV scale, devised by the MOS in the US based on the SF-20, was designed specifically for use in HIV-infected/AIDS patients. The scale evaluates 10 dimensions, including physical functioning, pain and discomfort, energy and fatigue, health distress, mental health, cognitive functioning, social functioning, role functioning, overall health, and quality of life, with a total of 35 items (**Supplementary Table S1**-**2** for MOS-HIV scoring details). Symptomomics Indicators: The following symptoms have been documented: cough, slow response, chills, lumbar pain, abdominal pain, loss of appetite, frequent urination, vomiting, Kaposi’s sarcoma, lymphadenopathy, fever, sore throat, diarrhea, infectious rash, herpes, and oral ulcers. Liver and Renal Function, Hematology Main Indicators: The following laboratory markers were assessed: red blood cells, hemoglobin, white blood cells, neutrophils, lymphocytes, creatinine, platelets, γ-glutamyl transpeptidase (GGT), aspartate aminotransferase (AST), blood urea nitrogen (BUN), and alanine aminotransferase (ALT). Immunological Markers: These following immune markers were measured: CD4, CD8, CD45RA+, CD45RO+, CD4CD25, CD4CD28, CD4CD38, CD8CD38, CD4Ki67/CD4 percentage, CD8Ki67/CD8, JC/CD4, and JC/CD8. Medication information was collected for all participants, including ART regimen composition, ART duration, medication adherence (self-report verified against pharmacy refill records), timing of the last dose before sample collection, and the use of statins, corticosteroids, traditional medicines, and other concomitant medications (**Supplementary Table S2-1**).

### 2.5 Plasma collection and analysis

Plasma was isolated from fresh blood samples by centrifugation and stored at –80 °C until analysis.

### 2.6 Metabolomic analysis

Thawed plasma samples were subjected to centrifugation at 600 × g for 5 min. The resulting supernatant was mixed with LC-MS grade methanol in a clean tube prior to analysis. The samples were maintained at –20 °C for 30 min and subjected to centrifugation at 4000 × g for 10 min at 20 °C to complete the precipitation process. The supernatants were transferred to new tubes and completely dried under nitrogen. The dried samples were dissolved in reconstitution solutions for sample injection into the ultra-performance liquid chromatography-tandem mass spectrometry (UPLC-MS/MS) systems. Untargeted metabolomic profiling of plasma samples from different groups was performed using an ACQUITY UPLC system (Waters, Milford, MA, USA) coupled to a Q Exactive mass spectrometer (Thermo Fisher Scientific) equipped with an ESI source. The chromatographic separation was achieved using a mobile phase comprised of 0.1% formic acid in water (solvent A) and 0.1% formic acid in acetonitrile (solvent B), with the column temperature maintained at 40 °C. The flow rate and injection volume were set at 0.25 mL/min and 2 µL, respectively.

The Mass spectrometric analysis was conducted using the following parameters: spray voltage, 3.5 kV and –2.5 kV for ESI (+) and ESI (–), respectively; sheath gas (nitrogen) flow rate, 30 arb; aux gas (nitrogen) flow rate, 10 arb; and capillary temperature, 325 °C. The full scan range was 100–1000 m/z with a resolution of 70000, and the MS/MS resolution was 17500. The normalized collision energy was set to 30 eV. OPLS-DA was conducted for p-values and variable importance projection (VIP). Fold change (FC) was used to identify variables contributing to classification. Statistically significant metabolites were identified as those with a VIP value > 1 and a p-value < 0.05. These differential metabolites (DMs) were then screened via MetaboAnalyst for further functional interpretation (Pang et al., 2024). The Kyoto Encyclopedia of Genes and Genomes (KEGG) Mapper tool was used to visualize the metabolites and corresponding pathways (Kanehisa et al., 2022). Drug- or diet-derived exogenous compounds (e.g., efavirenz, artemisinin, piperine, and caffeine metabolites) were excluded from downstream biological interpretation and KEGG pathway enrichment analyses. The confidence of metabolite identification was annotated according to the Metabolomics Standards Initiative (MSI) classification.

The metabolomic data reported in this paper have been deposited in the OMIX, China National Center for Bioinformation/Beijing Institute of Genomics, Chinese Academy of Sciences (https://ngdc.cncb.ac.cn/omix/preview/l3r8oeRg: accession no.OMIX014787).

### 2.7 Cytokine and chemokine multiplex analysis

Peripheral blood mononuclear cells (PBMCs) were isolated from fresh whole blood by Ficoll-Paque density gradient centrifugation according to the manufacturer’s instructions. As previously described, peripheral blood lymphocyte subsets were assessed by three-color flow cytometry using a Beckman Coulter Epics XL system (Bøyum, 1968). A panel of 48 inflammatory cytokines was quantified using a standardized Luminex assay (Bio-Rad Laboratories, Hercules, CA, USA) according to the manufacturer’s protocol. The panel comprised IL-1, IL-1RA, IL-2, IL-3, IL-4, IL-5, IL-6, IL-7, IL-8, IL-9, IL-10, IL-12, IL-13, IL-15, IL-16, IL-17A, and IL-18;tumor necrosis factor tumor necrosis factor (TNF)- and TNF-; Chemokines: Eotaxin, Growth-Regulated Oncogene-alpha, Leukemia Inhibitory Factor (LIF), Monocyte-Chemotactic Protein 3, Monokine induced by Gamma Interferon (MIG), Monocyte Chemoattractant Protein-1, crophage Inflammatory Protein-1 alpha, Macrophage Inflammatory Protein-1 beta, Cutaneous T-cell-Attracting Chemokine (CTACK). Regulated Upon Activation, Interferon-gamma (IFN-), Stromal Cell-Derived Factor 1 (SDF-1), Normal T-Cell Expressed and Presumably Secreted, Fibroblast Growth Factor, IFN-2, Platelet-Derived Growth Factor-BB (PDGF-BB), Granulocyte Colony-Stimulating Factor (CSF), Hepatocyte Growth Factor, Macrophage MIF, Stem Cell Factor, Stem Cell Growth Factor-beta, Beta-Nerve Growth Factor, Granulocyte-Macrophage CSF, Vascular Endothelial Growth Factor (VEGF), Macrophage CSF, and TNF-Related Apoptosis-Inducing Ligand (TRAIL).

### 2.8 Statistical methods

Statistical analysis and data visualization were both conducted employing the MetaboAnalyst platform (version 5.0), R statistical package (version 4.0.0; R Foundation for Statistical Computing, Vienna, Austria), and OriginPro 2023. Continuous variables were analyzed by Student’s t-test, and categorical variables by the chi-square test. Statistical significance was set at p < 0.05. Metabolome data were analyzed using the XCMS program for peak alignment, retention-time correction, and peak-area extraction. Metabolite structure identification and data pretreatment were conducted on the XCMS-extracted data, followed by subsequent quality control (QC) measures to evaluate the experimental data. Metabolite identification was performed by comparing the accuracy of the m/z values (< 25 ppm). Unsupervised principal component analysis (PCA) and supervised partial least squares discrimination analysis (PLS-DA) were performed to visualize sample clustering. The VIP value of each variable in the OPLS-DA model was calculated to indicate its contribution to the classification. Differentially expressed metabolites (DEMs) were defined by combining a VIP score > 1.0 from the PLS-DA model with a p-value < 0.05 from Welch’s t-test, as further visualized in volcano plots. The Benjamini–Hochberg procedure was used to control the false discovery rate (FDR < 0.05). DMs with fold change ≥ 2 and FC ≤ 0.5 were finally filtered as significantly altered metabolites. The normality of continuous variables was assessed using the Shapiro–Wilk test; variables that were not normally distributed were compared using the Mann–Whitney U test, and effect sizes (Cohen’s d or Hedges’ g) with 95% confidence intervals were reported for key comparisons. For the cytokine panel, group comparisons across the 42 analytes were adjusted for multiple testing using the Benjamini–Hochberg procedure, with FDR < 0.05 considered significant. PLS-DA models were validated by 200 permutation tests; R²Y, Q²Y, and permutation intercepts are reported for each pairwise model.

## 3 Results

This study systematically analyzed plasma lipid profiles and key biomarkers in fatigue and non-fatigue groups among HIV-infected individuals with INRs undergoing long-term ART using untargeted metabolomics and multiplex cytokine detection technologies. The research approach included sample collection, sample preparation, untargeted metabolomics analysis, multiplex cytokine detection, bioinformatics analysis, and validation of the key biomarkers (**Figure 1**).

### 3.1 Demographic characteristics

#### 3.1.1 Symptomatology and quality of life analysis

In this study, we performed a preliminary clinical characterization analysis involving HIV-infected INRs with (n = 18, mean fatigue score = 37.40 ± 5.49) and without fatigue (n = 17, mean fatigue score = 11.35 ± 9.97) (**Figure 2A**). Baseline characteristics, including age, sex, BMI, ART regimen, treatment duration, concomitant medications, and current immunological parameters (CD4 count, CD8 count, and CD4/CD8 ratio), are summarized in **Supplementary Tables S2-1** and **S2-2**. No statistically significant differences were observed between the INR-F and INR-NF groups for any variable (all *P* > 0.05). The results initially suggested significant clinical differences between fatigued and non-fatigued INRs undergoing long-term ART. Based on the MOS-HIV scale analysis, the fatigued group exhibited a significantly poorer quality of life than the non-fatigued group (**Figure 2B**). Further symptomomics analysis revealed that fatigued patients were more prone to exhibit symptom clusters, including loss of appetite, frequent urination, vomiting, lymphadenopathy, fever, sore throat, recurrent diarrhea, infectious rash, and oral ulcers (**Figure 2C**). Notably, one participant in the fatigued group (ID: 006042) was initially recorded with suspected Kaposi’s sarcoma, but this diagnosis was ruled out upon subsequent clinical follow-up and examination; therefore, no participant in either group had any AIDS-defining condition.

#### 3.1.2 Analysis of differences in blood routine, liver and kidney function, and immunity between the two groups

The results of routine blood tests provided a comprehensive analysis of variations in routine blood indices across subgroups of patients in the INR fatigue group. Notably, the red blood cell-related indices demonstrated the most significant differences among the subgroups (**Figure 2D**). Meanwhile, there were also notable trends of inter-group differences in indices such as white blood cell count, platelet count, and hemoglobin levels. Furthermore, an analysis of liver and kidney function indices in each subgroup revealed that the GGT index demonstrated the most prominent difference among subgroups (**Figure 2E**). Simultaneously, inter-group differences were observed in indices, including ALT, AST, BUN, and creatinine. Further analysis revealed that CD4 counts at enrolment (i.e., at sampling) did not differ significantly between the INR-F and INR-NF groups (**Figure 2F**).

### 3.2 Overview and distribution of metabolites

#### 3.2.1 Metabonomic quality control and overall analysis

In this study, metabolic analysis of INRs was performed using an HPLC-MS-based metabolomics approach. The 45 participants were divided into HCs (n = 10), fatigue (n = 18), and non-fatigue (n = 17) groups. During the analysis, 10 QC samples were used to assess the repeatability and stability of the pretreatment and instruments. Calculations were performed to obtain the relative standard deviation (RSD) values from the QC samples before and after batch correction for each metabolite. Of these, most metabolites (metabolites = 8392) exhibited an RSD below 20%, demonstrating excellent repeatability and stability. Using this strategy, we successfully screened hundreds of metabolites and metabolite sets, including amino acids and derivatives, lipids, carbohydrates, nucleotides and their derivatives, and others. Thus, it provides rich information for further investigation to understand the underlying metabolic mechanisms of INRs. Multivariate analysis (PCA and PLS-DA) revealed an association between the plasma metabolomic profile and fatigue in INRs (**Figures 3A, B**). The results indicated that all three groups exhibited a clear separation trend in metabolite distribution, particularly in the fatigue versus non-fatigue, fatigue versus health, and non-fatigue versus control groups. This indicates significant metabolic differences between patients and HCs, and fatigue may further influence the metabolic profiles of patients.

**Figure 3:**
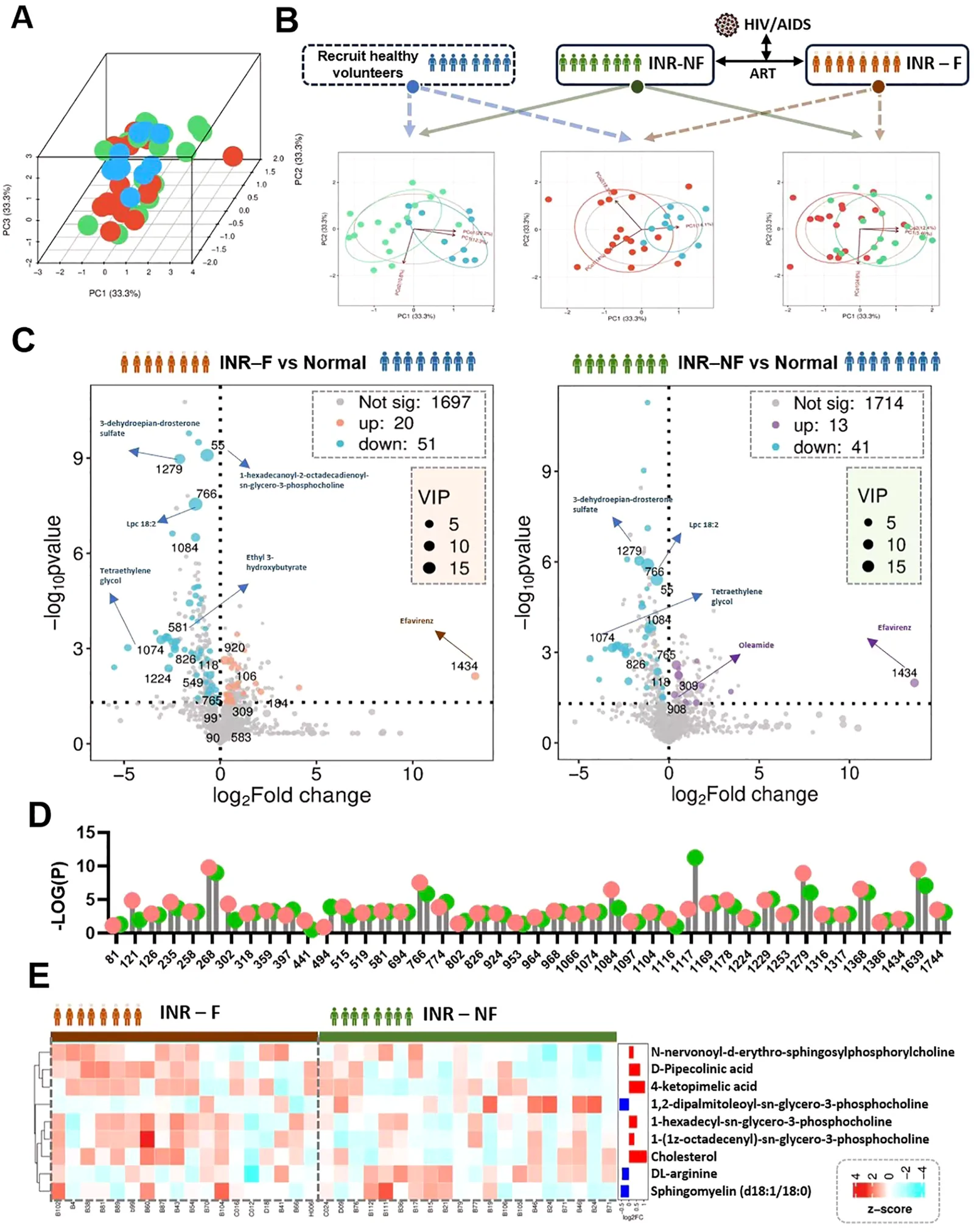
Overview and distribution of metabolites in the fatigue/non-fatigue population in INRs. **(A)**. Three-dimensional PCA plot illustrating the spatial distribution among different groups. **(B)** OPLS-DA score plot, with colors indicating distinct groups. **(C)** Volcano plots illustrating metabolite differences between the fatigue group and HCs, and between the non-fatigue group and HCs. **(D)** Lollipop plots displaying the log(p) values of shared differential metabolites between groups. **(E)** Representative differential metabolites. Heatmap of key differential metabolites between the fatigue and non-fatigue groups, highlighting prominent differences in cholesterol and sphingomyelin levels.

#### 3.2.2 Research results on the relationship between the INR fatigue population and metabolite spectrum

Furthermore, we screened for metabolites with large differences in content. The results indicated that 20 metabolites were significantly increased, whereas 51 metabolites were significantly reduced in the fatigue group compared with HCs (VIP > 1, p < 0.05). Compared with the HCs group, 13 metabolites were significantly increased, and 41 metabolites were significantly decreased in the non-fatigue group.

Notably, findings revealed that most metabolites were significantly up-regulated in the fatigue state (**Figure 3D**). For instance, efavirenz (ID: 1434) was elevated in the fatigued group relative to HCs (log2FC = 13.24, p = 0.01, and VIP = 4.56); however, a similarly large increase was observed in the non-fatigued INR group relative to HCs (log2FC = 13.60), and the direct comparison between the two INR groups showed no significant difference (P > 0.05). As both INR groups received ART whereas HCs did not, this signal reflects ART drug exposure rather than fatigue biology. Cholesterol (ID: 441) was up-regulated (log2FC = 1.84, p = 0.01, and VIP = 1.89), suggesting its accumulation in fatigued INR patients. Cis-9-Palmitoleic acid (ID: 1386), a lipid derivative, was also increased in tired INR patients (log2FC = 2.11, p = 0.02, and VIP = 2.44). Furthermore, certain lipid derivatives may accumulate during fatigue. Trans-3’-hydroxycotinine (ID: 1097) was also up-regulated during the fatigue state (log2FC = 4.09, p = 0.02, and VIP = 2.04), suggesting it may serve as a biomarker of fatigue in INRs. Furthermore, numerous metabolites were significantly decreased during the fatigue status of the INR. Piperine, paraxanthine (a caffeine metabolite), and artemisinin were likewise identified as exogenous, diet- or drug-derived compounds and were therefore excluded from all fatigue-related biological interpretation and pathway enrichment analyses. Moreover, ethyl 3-hydroxybutyrate (ID: 581) was decreased (log2FC = –2.75, p = 0.00, and VIP = 4.98), which further confirmed the inhibition of certain metabolic pathways during the fatigue state of INRs. The direct comparison between the fatigued (INR-F) and non-fatigued (INR-NF) groups was designated as the primary metabolomic analysis: the corresponding volcano plot is provided in **Supplementary Material 3**, and the complete statistics for all differential metabolites of this comparison (log2FC, raw and FDR-adjusted P values, VIP, fold change, and MSI identification level) are reported in **Supplementary Table S3-1**.

We identified significantly distinct metabolic patterns between fatigue and non-fatigue INRs using cluster analysis. During the fatigue state of INRs, the metabolites in the cluster were significantly up-regulated, including cholesterol and cis-9-Palmitoleic acid. The metabolites in the other cluster were down-regulated. This clustering pattern demonstrated a correlation between certain metabolites and fatigue status in INRs. The heatmap analysis suggested that the enriched metabolites exhibited significant differences between fatigue and non-fatigue INRs, including N-nervonoyl-d-erythro-sphingosylphosphorylcholine, D-pipecolinic acid, 4-ketopimelic acid, 1,2-dipalmitoleoyl-sn-glycero-3-phosphocholine, 1-hexadecyl-sn-glycero-3-phosphocholine, 1-(1z-octadecenyl)-sn-glycero-3-phosphocholine, cholesterol, DL-arginine, and sphingomyelin (**Figure 3F**).

#### 3.2.3 Analysis of the key KEGG pathways of the INR fatigue population

We identified 23 significantly enriched KEGG pathways by analyzing the up-regulated metabolites between the INR-F and Normal groups and 39 enriched signaling pathways between INR-NF and Normal groups. Amino acid metabolism, endocrine and metabolic diseases, and lipid metabolism were the important shared pathways in the KEGG enrichment analysis of the modules (**Figure 4A**). Furthermore, KEGG enrichment analysis of down-regulated metabolites revealed 28 enriched signaling pathways in the INR-F versus Normal group and 10 in the INR-NF versus Normal group. Among them, lysine degradation, protein digestion and absorption, vitamin digestion and absorption, and endocrine and metabolic diseases were key common pathways in the KEGG enrichment analysis of the modules (**Figure 4B**). The details of the different metabolic pathways are summarized in **Supplementary Tables S4**, **S5**.

**Figure 4:**
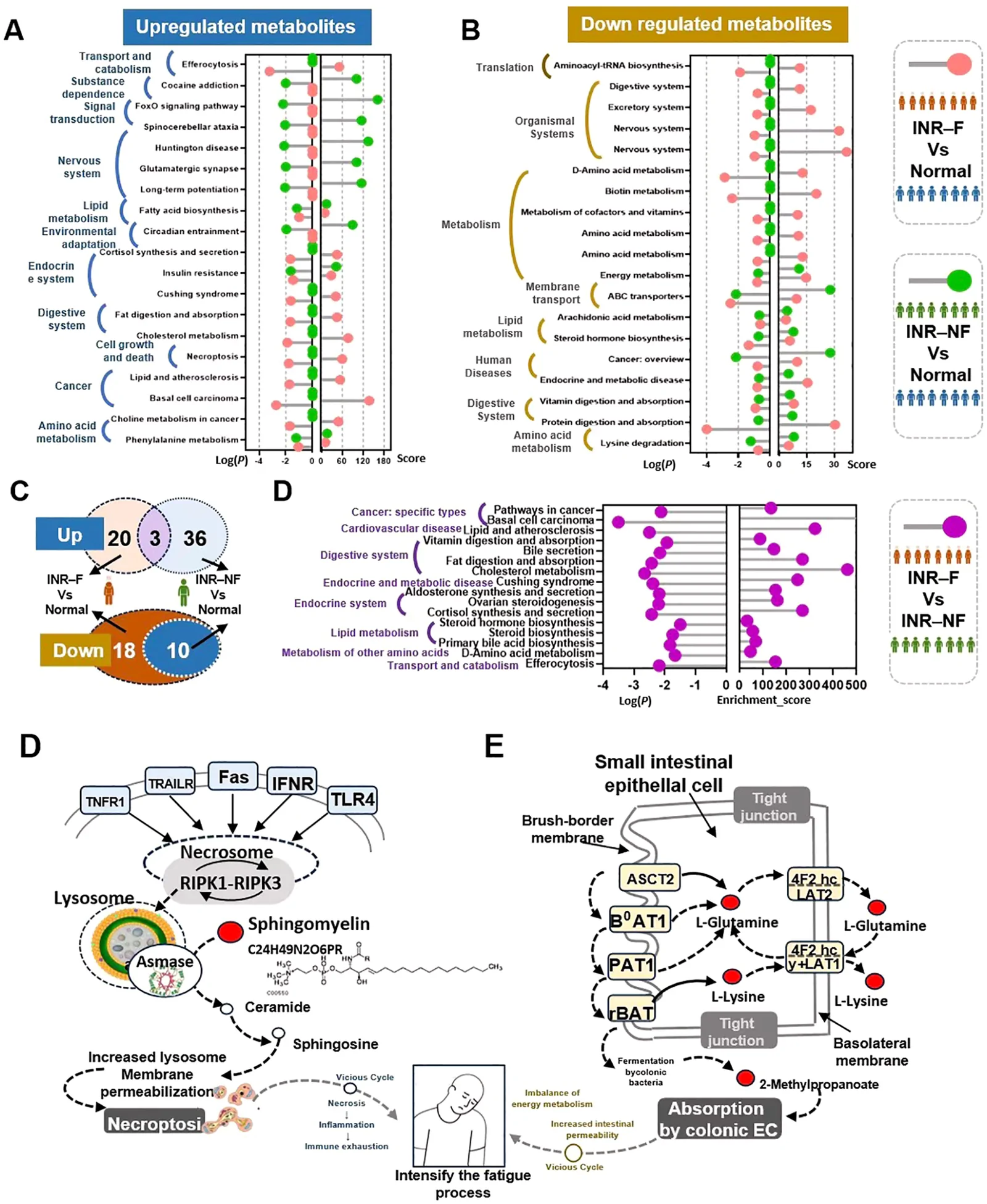
Fatigue and metabolite spectrum in INRs and KEGG critical pathway analysis. KEGG pathway analysis of differentially up-regulated metabolites in INR-F versus Normal and INR-NF versus Normal groups. **(B)** KEGG pathway analysis of differentially down-regulated metabolites in INR-F versus Normal and INR-NF versus Normal groups. **(C)** KEGG pathway analysis of up-regulated metabolites in INR-F versus INR-NF. **(D)** Representative example of an up-regulated pathway mechanism: enrichment-based inference of necroptosis pathway involvement (not experimentally measured). **(E)** Representative example of a down-regulated pathway mechanism: reduced protein digestion and absorption, proposed to contribute to pathology via three mechanisms, including energy depletion, immune suppression, and inflammation activation.

Furthermore, after exclusion of exogenous compounds, KEGG analysis of the direct comparison between the INR-F and INR-NF groups was performed and designated as the primary pathway analysis, revealing enrichment exclusively among up-regulated metabolites, with no enrichment of down-regulated pathways (**Figure 4C**). These pathways included efferocytosis, aldosterone synthesis and secretion, ovarian steroidogenesis, Cushing syndrome, cortisol synthesis and secretion, fat digestion and absorption, lipid metabolism and atherosclerosis, cholesterol metabolism, and basal cell carcinoma. Among them, enrichment of the necroptosis pathway (**Figure 4D**)—a pathway-level inference from enrichment analysis rather than direct experimental measurement—and reduced protein digestion and absorption (**Figure 4E**) may contribute to fatigue through impaired energy supply, immunosuppression, and inflammatory activation in INRs after ART; these hypotheses require experimental validation.

### 3.3 Cytokinomics

The multiplex detection system, an advanced analytical instrument that uses high-throughput detection technology, can concurrently perform quantitative analysis of over 50 biomarkers in a single sample using only a small volume of specimen (20–50 µL). In this study, the system was used to analyze samples from three groups and measure the expression levels of 42 cytokines. Subsequent statistical analyses were performed on the cytokines exhibiting significant differential expression, ultimately identifying 20 core differential cytokines. Specifically (Gazzola et al., 2009): In the comparison between the fatigued and health groups, the top differential cytokines (ranked by degree of difference) included IL-2RA, MIF, FGF, IFN-γ, IL-16, IL-1α, IL-8, IP-10, SDF-1α, CTACK, IL-18, GCSF, IL-1β, and TRAIL (**Figure 5A**) (Yang et al., 2020). In the comparison between non-fatigued and HC groups, the core differential cytokines (ranked by degree of difference) were IL-2RA, IL-16, IL-18, IP-10, MCSF, IFN-α2, LIF, IL-1α, FGF, IL-1RA, MIG, eotaxin, IL-1β, MIF, and IFN-γ (**Figure 5B**). Furthermore, protein-protein interaction (PPI) network analysis was performed across the different groups (**Figure 5C**). Through visualization analysis, the patterns of differential cytokine expression in each group were examined. The analysis revealed that key cytokines, including IL-18, IP-10, CXCL8, IL-1α, and TNF-α, interacted strongly with multiple metabolism-related proteins.

**Figure 5:**
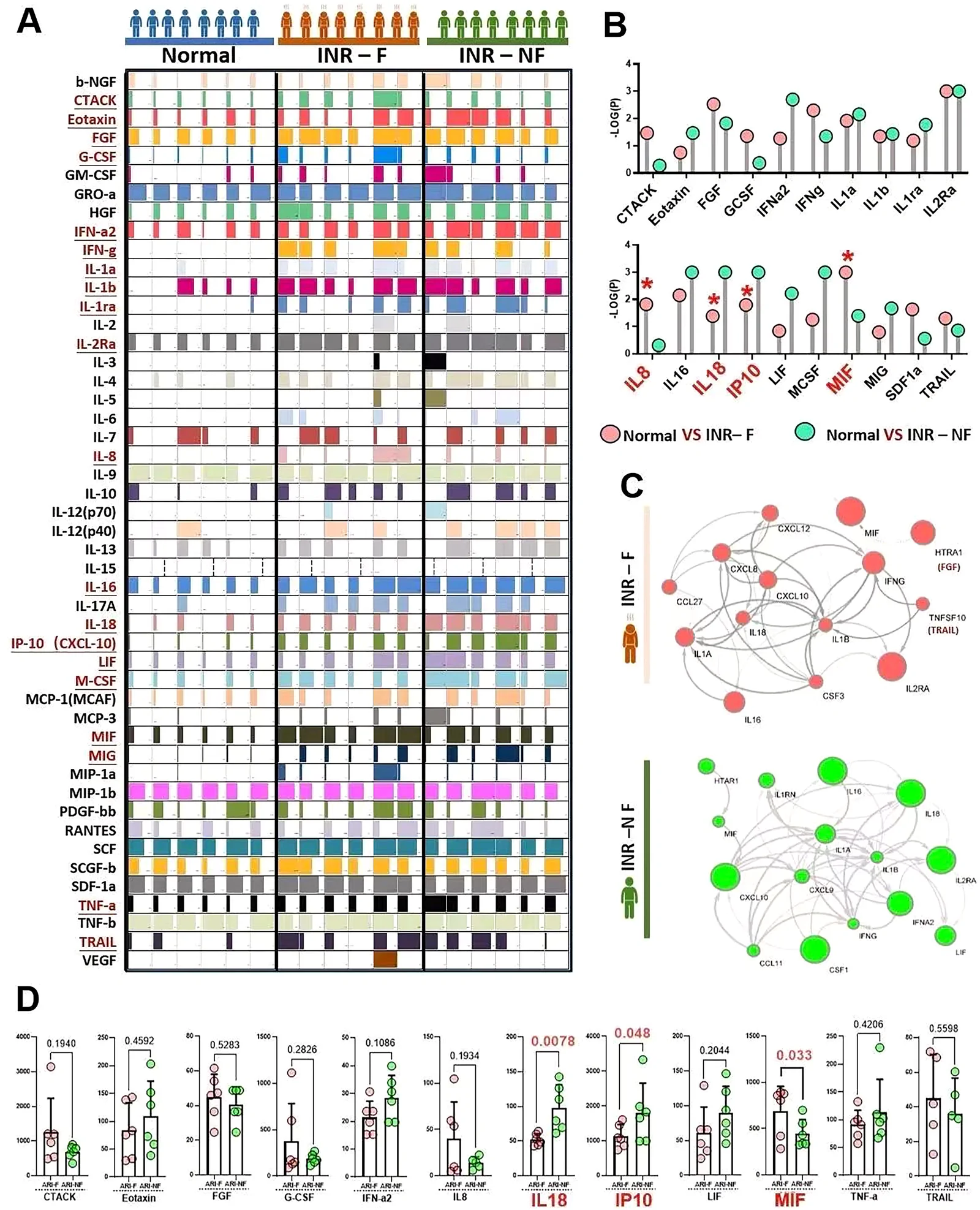
Identification of potential biomarkers of fatigue in INRs by multi-factor detection (Cytokinomics). **(A)** Heatmap of 42 cytokinomics, with normalized expression proportions for each cytokine. Cytokine names represented in red font indicate statistically significant differences by analysis of variance (p < 0.05). **(B)** Further analysis of significantly differentially expressed cytokines identified 20 core differential cytokines. **(C)** PPI network analysis of different groups. **(D)** Validation of differential targets between the fatigued and non-fatigued groups revealed three significantly different targets (ranked by decreasing significance): IL-18 (p < 0.01), IP-10, and MIF, * indicates the core cytokines of interest.

Furthermore, validation analysis was performed on differential targets between the fatigued and non-fatigued groups (**Figure 5D**) and identified three targets that differed significantly between INRs with and without fatigue (ranked from highest to lowest difference): IL-18 (p < 0.01), IP-10, and MIF. IL-18, a core pro-inflammatory cytokine involved in T helper cell (Th1)-type immune responses, was down-regulated in the fatigued group (lower than in the non-fatigued group), potentially indicating an “exhaustive suppression” of the immune system in response to persistent inflammation. However, its level remained higher than that of the HC group, implying that INRs experience an incompletely resolved state of chronic, low-grade inflammation. This “insufficient suppression” may result in fatigue by disrupting the energy metabolism and neurotransmitter balance. IP-10 (CXCL10), an important chemokine that recruits T-cells and natural killer cell (NK) cells, was decreased in the fatigued group compared to the non-fatigued group, potentially signifying impaired immune cell migration ability, which may diminish the efficiency of viral reservoir clearance. However, its level remained elevated compared to that of HCs, suggesting that patients were in an “immune-activated but functionally impaired” intermediate state. These contradictory signals may induce fatigue by continuously depleting immune resources. This interesting situation suggests that the decrease in inflammatory cytokines in the fatigued group may present an “adaptive down-regulation” of the immune system in response to persistent stimulation to avoid excessive damage. However, this down-regulation may still be associated with functional deficiencies that are significantly higher than those in HCs.

### 3.4 Analysis of potential biomarkers based on the relationship between metabolites and inflammatory cytokines

To further explore the complex molecular mechanisms underlying fatigue in INR patients, we focused on the interactions between metabolomics and cytokines, given their interconnected and synergistic roles in physiological and pathological processes. These two factors may be closely related at the signaling pathway level, collectively affecting disease progression and the immune status. In an innovative approach, we used a combined analysis strategy that integrated KEGG enrichment analysis of both the metabolome and cytokines. Using a Venn diagram integrated with literature tracing, we visually depicted the overlap between the two, aiming to comprehensively and systematically identify the key signaling pathways and related molecules associated with fatigue in INR patients.

The combined analysis results demonstrated that three key signaling pathways were jointly enriched in the Venn diagram overlap between the metabolomics and cytokine protein KEGG pathways for the fatigued and normal groups (**Figures 6A, B**) (Gazzola et al., 2009). Necroptosis Pathway: pathway enrichment analysis of the plasma metabolomic and cytokine data suggested potential involvement of the necroptosis pathway in fatigued INR patients. It should be emphasised that no experimental assays (e.g., Western blot or qPCR) of necroptosis marker expression in PBMCs were performed in this study; this enrichment-based inference raises the hypothesis that necroptosis may accelerate immune cell death, further impair immune reconstitution, and thereby contribute to the onset and progression of fatigue in INR patients—a hypothesis that requires experimental validation (Yang et al., 2020). Lipid and Atherosclerosis Pathway: Studies have demonstrated that INR patients exhibit a 2–3 times higher risk of developing cardiovascular diseases than those with normal immune reconstruction, and their arterial plaques tend to be more unstable, rendering them more prone to acute cardiovascular events. The enrichment of this pathway may provide a molecular explanation for the elevated incidence of cardiovascular diseases and plaque instability observed in INR patients (Henderson et al., 2005). Pathways in Cancer: Several cancer-related pathways in INR patients, such as those involved in PI3K-AKT and WNT/β-catenin crosstalk, were overactivated. These pathways are involved in tumorigenesis and are closely associated with immune escape mechanisms, suggesting that INR patients may be at increased risk of tumor development and immune system dysfunction.

**Figure 6:**
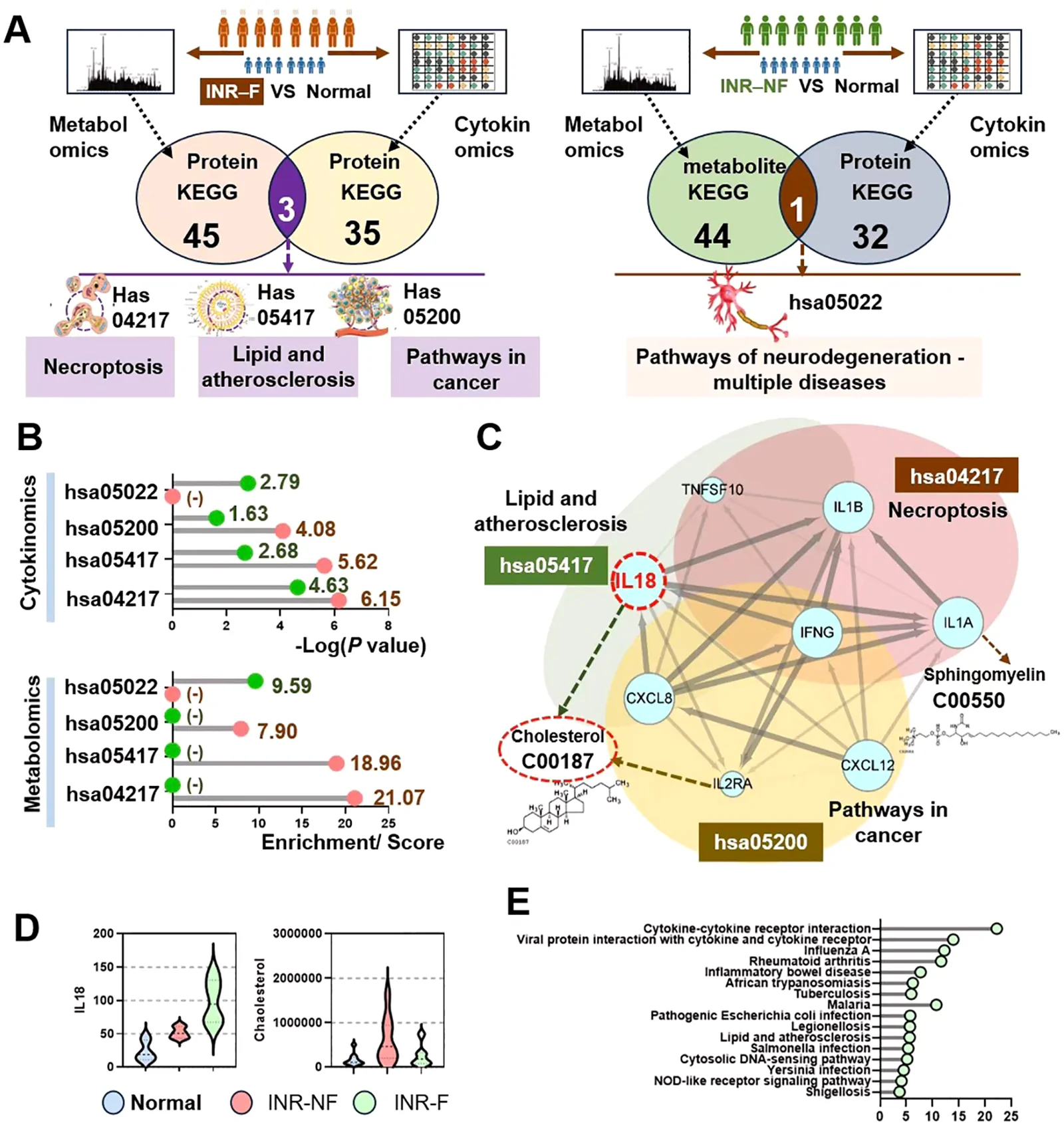
Mining potential biomarkers based on association analysis of metabolites and cytokines in KEGG. **(A)** Venn diagram illustrating the intersection of KEGG-enriched pathways from metabolomic and cytokine (proteomic) analyses between the fatigue group and HCs, identifying three key signaling pathways: necroptosis, lipid and atherosclerosis, and pathways in cancer. **(B)** Lollipop chart illustrating the expression scores of key signaling pathways across different metabolomic and cytokine datasets. **(C)** Protein-metabolite interaction network construction identified key factors in INRs, confirming sphingomyelin and cholesterol as core metabolic biomarkers in the fatigued INR group. Among them, IL-18 and cholesterol were identified as candidate markers associated with fatigue in INRs. **(D)** Quantitative comparison of IL-18 and cholesterol expression across different groups. **(E)** KEGG pathway analysis demonstrating IL-18-related signaling processes following ART.

To explore the critical factors of INR, we constructed a protein-metabolite network (**Figure 6C**). Using this network, we identified a series of key factors, including cytokines such as IFN-γ, IL-1α, IL-1β, TNFSF10, CXCL8, IL-18, IL-2RA, and CXCL12, as well as metabolic markers such as sphingomyelin and cholesterol. There was no significant association between IL-18 concentration and cholesterol levels in any of the three groups; only a non-significant positive trend was observed in INR-F patients. The specific mechanisms and clinical significance of these trends require further investigation.

In summary, through network inference and correlation analysis, we identified IL-18 and cholesterol as candidate markers associated with fatigue in the INR population. Given that their correlation did not reach statistical significance and that discrimination metrics (e.g., AUC, sensitivity, and specificity) were not evaluated, their combined value as a biomarker panel remains exploratory and requires validation in independent, larger cohorts.

## 4 Discussion

Previous studies have demonstrated that fatigue is highly prevalent among individuals with HIV and significantly impairs their quality of life. However, in the specific subgroup of INRs, the characteristics, associated biomarkers, and underlying pathogenic mechanisms remain poorly understood. This study focused on fatigue in HIV-INRs undergoing ART. By integrating clinical characteristics, untargeted metabolomics, and high-throughput cytokine profiling, we examined the potential biological basis of fatigue syndrome in ART-treated INRs. Our findings revealed that the INR-fatigued population exhibited worse clinical outcomes and displayed significantly distinct plasma metabolite and cytokine profiles compared with their non-fatigued counterparts. Cholesterol and IL-18 were identified as candidate markers associated with fatigue status, although their combined discriminatory performance was not statistically validated. These results provide a novel multi-omics perspective for understanding the pathophysiological mechanisms underlying fatigue in INRs.

This study demonstrated that, in addition to the primary symptom of fatigue, the fatigued INRs group exhibited a more pronounced decrease in plasma lipid metabolites, consistent with previous studies (Thangaraj et al., 2018; Nass et al., 2023; Elahi et al., 2024). These findings suggest that fatigue often coexists with various physiological and psychological manifestations, forming a symptom cluster that significantly diminishes quality of life. Moreover, the fatigued group exhibited significant concomitant reductions in red blood cell count, GGT levels, lymphocyte count, and CD4+ T-cell count. The immunodeficient state reflected by these clinical manifestations and laboratory indicators provides valuable preliminary insights into the immunological basis of fatigue in INRs.

Untargeted metabolomic analysis revealed that lipid metabolites accounted for the largest proportion of differentially expressed molecules, highlighting the central role of lipid metabolism in fatigue in INRs. Among these, significantly elevated plasma cholesterol levels were one of the most critical findings. Pathway enrichment analysis revealed enrichment of the “Aldosterone synthesis and secretion” and “Cushing syndrome” pathways, which may point to involvement of the hypothalamic-pituitary-adrenal (HPA) axis; as the core system for stress response, its dysregulation has been linked to chronic fatigue, sleep disturbance, and cognitive impairment. Given that steroidogenesis-related pathways are influenced by sex, the sex distribution of the two groups (**Supplementary Table S2-1**) was taken into account when interpreting these pathways. Moreover, as cholesterol is a common precursor of all adrenal corticosteroids, its elevation may partly drive the enrichment of these pathways; therefore, any HPA-axis involvement inferred from this cross-sectional snapshot should be regarded as a suggestive finding that requires confirmation in longitudinal and experimental studies.

Consistent with our findings, a cross-sectional study on HDL-C abnormalities and severe fatigue in major depressive disorder (MDD) indicated that HDL-C may protect patients with MDD from severe fatigue (Tseng et al., 2024). Our findings suggest that certain lipids, particularly LDL-C and apolipoproteins E and B, contribute to CFS development. Conversely, HDL-C and apolipoprotein A1 may delay the onset of this syndrome, with immune cells mediating lipid-mediated pathways that affect CFS. Elevated cholesterol levels may exacerbate fatigue by promoting chronic inflammation and immune activation, affecting cell membrane structure, lipid raft signaling, and immune cell metabolic states. Therefore, dyslipidemia is a core metabolic feature of INR-associated fatigue, potentially serving as a critical basis for disease susceptibility and clinical severity.

Cytokine analysis revealed a complex pattern of immune dysregulation. Patients in the fatigue group exhibited increased plasma MIF levels, whereas IL-18 and IP-10 levels, although lower than those in the non-fatigue group, remained significantly higher than those in HCs. Elevated levels of MIF, a multifunctional pro-inflammatory cytokine, suggest a persistent inflammatory response. The most intriguing finding concerns IL-18 and IP-10 (CXCL10) levels; although lower than those in the non-fatigued INR group, they remained significantly higher than those in HCs. This “intermediate state” reveals a key mechanism: fatigue probably arises not from absolute increases or decreases in specific inflammatory factors but from an imbalance in immune regulatory function. A clinical study treating long COVID with PTIC injections also observed that the down-regulation of MIF, IL-18, and IP-10 was associated with chronic fatigue syndrome in patients. PPI network and KEGG pathway analyses indicated that these factors are primarily involved in inflammatory and metabolic signaling pathways, including necroptosis (a pathway-level inference from enrichment analysis rather than an experimental measurement), lipid metabolism, atherosclerosis, and cancer.

Combined metabolomics and high-throughput cytokine analysis revealed no significant association between IL-18 and cholesterol levels in any group (HCs: p = 0.91; INR-F: R = 0.478, p = 0.128; INR-NF: p = 0.998); only a non-significant positive trend was observed in INR-F patients, which requires verification in larger samples. Therefore, cholesterol and IL-18 are not merely differential molecules but are core elements spanning the entire pathophysiological process. Operating at the metabolic and immune levels, they form a vicious cycle of “metabolism-cell death-inflammation,” potentially serving as a key mechanism driving persistent fatigue (Olivares-Martinez et al., 2025). Accordingly, the combined use of cholesterol and IL-18 should be regarded as an exploratory finding pending independent validation, rather than a validated tool for clinical classification or risk prediction.

This study has several limitations. The relatively small sample size and single-center design may limit the generalizability of our findings. Furthermore, its cross-sectional design precludes causal inference between metabolic and cytokine alterations and fatigue, and although medication information was collected and compared between the two groups (**Supplementary Table S3-2**), residual confounding by ART regimen—particularly efavirenz exposure—cannot be fully excluded given the limited sample size (17–18 participants per group), and other potential confounders, such as comorbid metabolic disorders and psychosocial factors, were not fully addressed. In addition, the fatigued group at ART initiation showed lower CD4+ counts, so pre-ART disease severity remains a competing explanation for the observed molecular differences, even though no participant had any AIDS-defining condition (**Supplementary Material 2**). The modified FACIT-FS used in this study has not undergone formal psychometric validation; the 30-point threshold was additionally evaluated by ROC analysis against the MOS-HIV energy/fatigue dimension (**Supplementary Material**). Accordingly, all findings of this study should be considered exploratory and hypothesis-generating, pending validation in independent cohorts. Current multi-omics coverage is also limited, as transcriptomics, proteomics, and gut microbiome data are not incorporated, potentially neglecting critical pathways. Moreover, the mechanistic roles of cholesterol and IL-18 require experimental validation. Future studies should use large-scale, multicenter, longitudinal, and interventional designs, integrating multi-omics layers to elucidate the metabolic-immune network underlying fatigue systematically. Whether the cholesterol-IL-18 axis can inform predictive models or therapeutic strategies for risk stratification and personalized management of HIV-INRs remains to be established in such future studies.

## 5 Conclusion

This study demonstrates that HIV-INRs with fatigue exhibit distinctive clinical and biological signatures, including lower red blood cell and CD4+ T-cell counts, lipid metabolic disturbances, and cytokine imbalances. Elevated cholesterol and increased MIF, along with intermediate IL-18 and IP-10 levels, indicate disrupted Th1 immunity and an interplay between metabolism and inflammation. Pathway enrichment analysis suggested—at the level of pathway enrichment inference rather than experimental measurement—convergence on necroptosis and lipid-driven inflammation, supporting a potential vicious cycle sustaining fatigue. Notably, cholesterol and IL-18 emerged as candidate biomarkers whose combined assessment showed only an exploratory, non-validated association with fatigue status. **Figure 7** illustrates the mechanism. These findings provide novel insights into the pathophysiology of fatigue in patients undergoing ART and suggest that integrating metabolic and immune markers may support risk stratification and personalized management.

**Figure 7:**
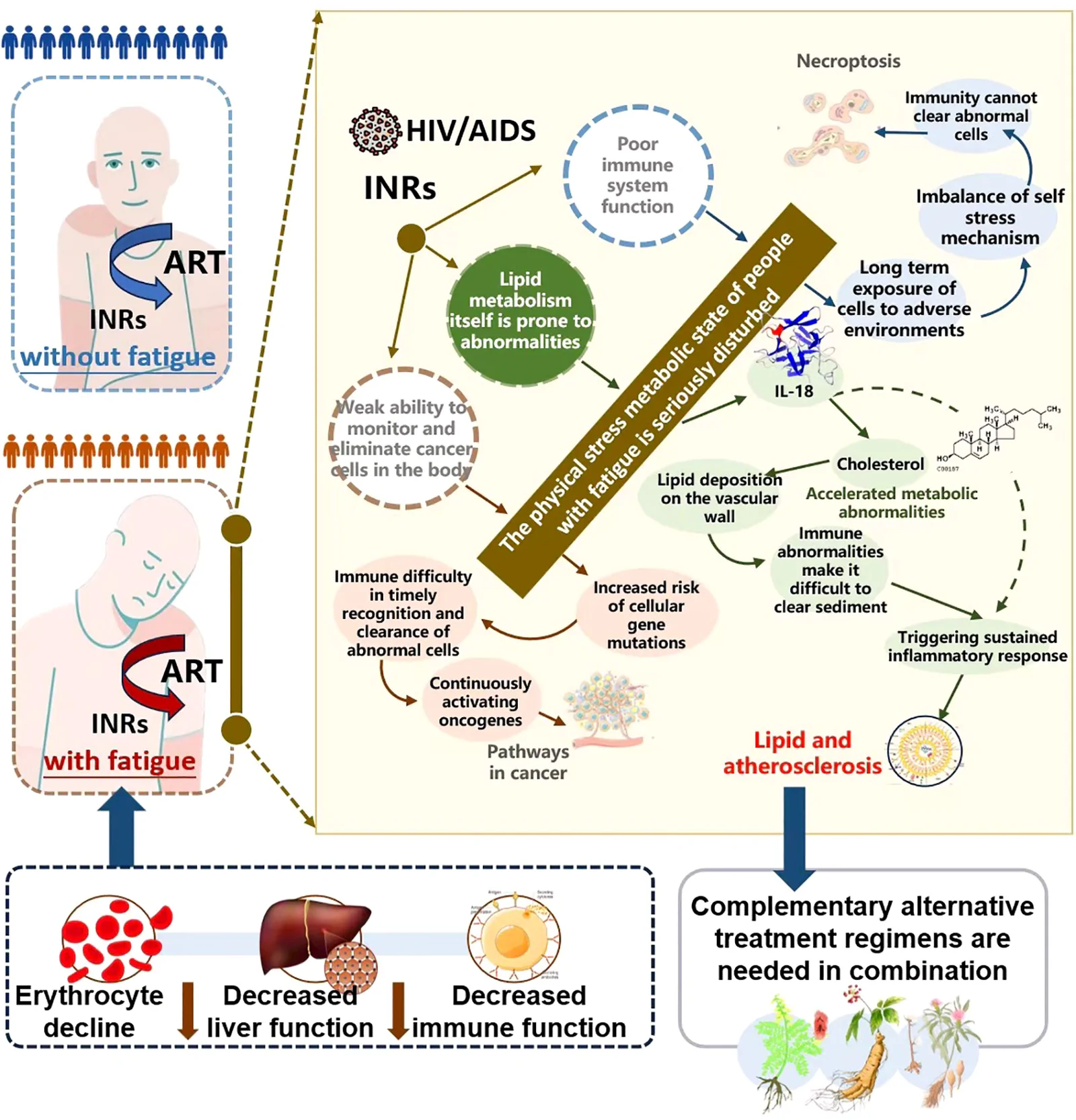
Mechanism summary chart. Abnormal activation of “Lipid and atherosclerosis”: Incomplete immune reconstitution → altered IL-18 levels (key biomarker) → predisposition to lipid metabolic abnormalities (for instance, disrupted cholesterol and related lipid metabolism) → functional decline due to poor physical status in fatigued individuals → weakened lipid regulation capacity → increased cholesterol and lipid deposition on vascular walls → impaired immune clearance of lipid deposits due to immune dysfunction → persistent lipid-induced inflammatory response (involving IL-18 and other inflammatory cytokines) → abnormal activation of “Lipid and atherosclerosis” pathway.

## Data Availability

The datasets presented in this study can be found in online repositories. The names of the repository/repositories and accession number(s) can be found below: https://ngdc.cncb.ac.cn/omix/preview/l3r8oeRg, OMIX014787.
