# Supplementary material for: Integrated multi-omics reveals plasma biomarkers of fatigue in HIV immunological non-responders
Source: Front Cell Infect Microbiol. 2026 Oct 15;16:1932594. doi: 10.3389/fcimb.2026.1932594 (PMC13716617; doi:10.3389/fcimb.2026.1932594)
Supplement: Supplementary file 1 [file Table1.docx]

Supplementary Material 1

# Supplementary Tables

**Supplementary Tables**

**Table S1-1. Functional assessment of chronic illness therapy–fatigue (FACIT-F) scale.**

| **Q** | **Statement** | **Not at all** | **A little bit** | **Somewhat** | **Quite a bit** | **Very much** |
| --- | --- | --- | --- | --- | --- | --- |
| An1 | I feel very fatigued | 0 | 1 | 2 | 3 | 4 |
| An2 | I feel weak all over | 0 | 1 | 2 | 3 | 4 |
| An3 | I feel listless | 0 | 1 | 2 | 3 | 4 |
| An4 | I feel tired | 0 | 1 | 2 | 3 | 4 |
| An5 | I can't get going because I'm tired | 0 | 1 | 2 | 3 | 4 |
| An6 | I can't finish things because I'm tired | 0 | 1 | 2 | 3 | 4 |
| An7 | I have energy | 0 | 1 | 2 | 3 | 4 |
| An8 | I am able to do my usual activities | 0 | 1 | 2 | 3 | 4 |
| An9 | I need to sleep during the day | 0 | 1 | 2 | 3 | 4 |
| An10 | I am too tired to eat | 0 | 1 | 2 | 3 | 4 |
| An11 | I need help doing my usual activities | 0 | 1 | 2 | 3 | 4 |
| An12 | I feel frustrated because I'm too tired to do the things I want to do | 0 | 1 | 2 | 3 | 4 |
| An13 | I have to limit my social activity because I'm tired | 0 | 1 | 2 | 3 | 4 |
| An14 | Antiretroviral medications (ART) contribute to my fatigue | 0 | 1 | 2 | 3 | 4 |
| An15 | Fatigue makes it harder to adhere to my HIV treatment regimen | 0 | 1 | 2 | 3 | 4 |

This version maintains the structure and content of the original simplified Chinese version, with each statement followed by a scale from 0 (Not at all) to 4 (Very much) for patients to rate their level of agreement or severity of the symptom. This scale is commonly used to assess fatigue severity in patients with chronic illnesses, including those undergoing long-term ART and chronic fatigue syndrome (ME/CFS).

**Table S1-2. MOS-HIV questionnaire.**

| **Question** | **Options** |
| --- | --- |
| **1. Overall, how would you rate your health?** | Please check one: |
|  | Excellent □ |
|  | Very Good □ |
|  | Good □ |
|  | Fair □ |
|  | Poor □ |
| **2. In the past four weeks, have you had any bodily pain?** | Please check one: |
|  | No pain at all □ |
|  | Very mild pain □ |
|  | Mild pain □ |
|  | Moderate pain □ |
|  | Severe pain □ |
|  | Very severe pain □ |
| **3. In the past four weeks, has bodily pain interfered with your normal work (including both paid work and housework)?** | Please check one: |
|  | Not at all □ |
|  | A little bit □ |
|  | Moderately □ |
|  | Quite a bit □ |
|  | Extremely □ |
| **The following questions relate to daily activities. Has your health limited these activities? If so, to what extent?** | |
| **4. Strenuous activities (e.g., lifting heavy objects, running, vigorous sports)** | Not limited at all □ |
|  | Limited a little □ |
|  | Limited a lot □ |
| **5. Moderate activities (e.g., moving a table, carrying groceries, doing exercises)** | Not limited at all □ |
|  | Limited a little □ |
|  | Limited a lot □ |
| **6. Walking uphill or climbing several flights of stairs** | Not limited at all □ |
|  | Limited a little □ |
|  | Limited a lot □ |
| **7. Bending, kneeling, or stooping** | Not limited at all □ |
|  | Limited a little □ |
|  | Limited a lot □ |
| **8. Walking a distance of about 100 meters** | Not limited at all □ |
|  | Limited a little □ |
|  | Limited a lot □ |
| **9. Eating, dressing, bathing, or using the toilet** | Not limited at all □ |
|  | Limited a little □ |
|  | Limited a lot □ |
| **10. Has your health kept you from working, doing housework, or going to school?** | Please check one: |
|  | Yes □ |
|  | No □ |
| **11. Has your health kept you from doing certain parts of your work, housework, or studies?** | Please check one: |
|  | Yes □ |
|  | No □ |
| **For the following questions, please select the answer that most closely matches how you have felt during the past four weeks.** | |
| **12. Your health has limited your social activities (e.g., visiting friends or relatives)** | All of the time □ |
|  | Most of the time □ |
|  | A good bit of the time □ |
|  | Some of the time □ |
|  | A little of the time □ |
|  | None of the time □ |
| **13. You are a nervous person** | All of the time □ |
|  | Most of the time □ |
|  | A good bit of the time □ |
|  | Some of the time □ |
|  | A little of the time □ |
|  | None of the time □ |
| **14. You feel calm** | All of the time □ |
|  | Most of the time □ |
|  | A good bit of the time □ |
|  | Some of the time □ |
|  | A little of the time □ |
|  | None of the time □ |
| **15. You feel downhearted** | All of the time □ |
|  | Most of the time □ |
|  | A good bit of the time □ |
|  | Some of the time □ |
|  | A little of the time □ |
|  | None of the time □ |
| **16. You are a happy person** | All of the time □ |
|  | Most of the time □ |
|  | A good bit of the time □ |
|  | Some of the time □ |
|  | A little of the time □ |
|  | None of the time □ |
| **17. You feel so discouraged that nothing can cheer you up** | All of the time □ |
|  | Most of the time □ |
|  | A good bit of the time □ |
|  | Some of the time □ |
|  | A little of the time □ |
|  | None of the time □ |
| **18. You feel full of energy** | All of the time □ |
|  | Most of the time □ |
|  | A good bit of the time □ |
|  | Some of the time □ |
|  | A little of the time □ |
|  | None of the time □ |
| **19. You feel exhausted** | All of the time □ |
|  | Most of the time □ |
|  | A good bit of the time □ |
|  | Some of the time □ |
|  | A little of the time □ |
|  | None of the time □ |
| **20. You feel fatigued** | All of the time □ |
|  | Most of the time □ |
|  | A good bit of the time □ |
|  | Some of the time □ |
|  | A little of the time □ |
|  | None of the time □ |
| **21. You have enough energy to do the things you want to do** | All of the time □ |
|  | Most of the time □ |
|  | A good bit of the time □ |
|  | Some of the time □ |
|  | A little of the time □ |
|  | None of the time □ |
| **22. You feel weighed down by your health problems** | All of the time □ |
|  | Most of the time □ |
|  | A good bit of the time □ |
|  | Some of the time □ |
|  | A little of the time □ |
|  | None of the time □ |
| **23. You feel disheartened because of your health problems** | All of the time □ |
|  | Most of the time □ |
|  | A good bit of the time □ |
|  | Some of the time □ |
|  | A little of the time □ |
|  | None of the time □ |
| **24. You feel hopeless because of your health problems** | All of the time □ |
|  | Most of the time □ |
|  | A good bit of the time □ |
|  | Some of the time □ |
|  | A little of the time □ |
|  | None of the time □ |
| **25. You feel afraid because of your health problems** | All of the time □ |
|  | Most of the time □ |
|  | A good bit of the time □ |
|  | Some of the time □ |
|  | A little of the time □ |
|  | None of the time □ |
| **26. You have difficulty thinking or solving problems (e.g., making plans, making decisions, learning new things)** | All of the time □ |
|  | Most of the time □ |
|  | A good bit of the time □ |
|  | Some of the time □ |
|  | A little of the time □ |
|  | None of the time □ |
| **27. You forget recent events (e.g., where you put things, appointment times)** | All of the time □ |
|  | Most of the time □ |
|  | A good bit of the time □ |
|  | Some of the time □ |
|  | A little of the time □ |
|  | None of the time □ |
| **28. You have difficulty concentrating during activities that require sustained attention** | All of the time □ |
|  | Most of the time □ |
|  | A good bit of the time □ |
|  | Some of the time □ |
|  | A little of the time □ |
|  | None of the time □ |
| **29. You have difficulty doing activities that require focus and thought** | All of the time □ |
|  | Most of the time □ |
|  | A good bit of the time □ |
|  | Some of the time □ |
|  | A little of the time □ |
|  | None of the time □ |
| **The following statements describe how one might feel about their health. Please select the answer that most closely matches your situation.** | |
| **30. My body feels a bit uncomfortable** | Definitely true □ |
|  | Mostly true □ |
|  | Not sure □ |
|  | Mostly false □ |
|  | Definitely false □ |
| **31. I am as healthy as anyone I know** | Definitely true □ |
|  | Mostly true □ |
|  | Not sure □ |
|  | Mostly false □ |
|  | Definitely false □ |
| **32. My health is excellent** | Definitely true □ |
|  | Mostly true □ |
|  | Not sure □ |
|  | Mostly false □ |
|  | Definitely false □ |
| **33. I have been feeling very bad lately** | Definitely true □ |
|  | Mostly true □ |
|  | Not sure □ |
|  | Mostly false □ |
|  | Definitely false □ |
| **34. In the past four weeks, how would you rate your quality of life? That is, have you felt that everything is going smoothly?** | Couldn't be better □ |
|  | Very good □ |
|  | Mixed □ |
|  | Bad □ |
|  | Couldn't be worse □ |
| **35. Compared to four weeks ago, how would you rate your current physical health and emotional well-being?** | Much better □ |
|  | A little better □ |
|  | About the same □ |
|  | A little worse □ |
|  | Much worse □ |

**ROC Analysis of the Modified FACIT-FS Threshold Against the MOS-HIV Energy/Fatigue Dimension**

To evaluate the discriminatory performance of the modified 15-item FACIT-FS threshold (≥30 vs. <30), we performed a receiver operating characteristic (ROC) analysis using the Energy/Fatigue dimension (Q18–Q21) of the MOS-HIV as the reference standard. The MOS-HIV energy/fatigue dimension comprises four items scored on a 6-point Likert scale (total range 4–24), with higher scores reflecting worse energy/fatigue status. Because no universally accepted fatigue threshold exists for this dimension, we applied a median split (median = 15) to dichotomise the 35 participants into "fatigue" (score ≥15, n = 18) and "non-fatigue" (score <15, n = 17) groups, yielding group sizes nearly identical to the original FACIT-FS classification. The two instruments showed strong concordance: 31 of 35 participants (88.6%) were classified identically by both instruments. FACIT-FS total scores correlated strongly with MOS-HIV energy/fatigue scores (Spearman ρ = 0.716, P < 0.001). The AUC for FACIT-FS was 0.964 (95% CI: 0.897–1.000, bootstrap n = 2,000), indicating excellent discrimination. Against the MOS-HIV reference standard, the pre-specified FACIT-FS cut-off of 30 achieved a sensitivity of 0.889 (16/18), specificity of 0.882 (15/17), positive predictive value of 0.889, negative predictive value of 0.882, and overall accuracy of 88.6% (31/35). The Youden-index-derived optimal cut-off was 25 (sensitivity 1.000, specificity 0.824, Youden index 0.824); however, the 30-point threshold provides a more balanced sensitivity–specificity profile and remains a clinically pragmatic choice for this exploratory study. All analyses were conducted in Python (v3.12) with scikit-learn and SciPy.

| **Participant ID** | **Original Group** | **FACIT-FS Score** | **MOS-HIV Energy/Fatigue Score** | **MOS-HIV Classification** |
| --- | --- | --- | --- | --- |
| 6001 | Fatigued | 39 | 19 | Fatigue |
| 6002 | Non-fatigued | 7 | 11 | Non-fatigue |
| 6006 | Fatigued | 40 | 19 | Fatigue |
| 6007 | Fatigued | 35 | 17 | Fatigue |
| 6008 | Fatigued | 32 | 17 | Fatigue |
| 6015 | Fatigued | 37 | 18 | Fatigue |
| 6016 | Fatigued | 40 | 20 | Fatigue |
| 6017 | Non-fatigued | 3 | 13 | Non-fatigue |
| 6018 | Fatigued | 36 | 17 | Fatigue |
| 6019 | Non-fatigued | 5 | 12 | Non-fatigue |
| 6023 | Non-fatigued | 14 | 11 | Non-fatigue |
| 6025 | Fatigued | 39 | 19 | Fatigue |
| 6026 | Fatigued | 38 | 18 | Fatigue |
| 6028 | Non-fatigued | 2 | 13 | Non-fatigue |
| 6029 | Fatigued | 36 | 17 | Fatigue |
| 6032 | Fatigued | 42 | 20 | Fatigue |
| 6033 | Non-fatigued | 18 | 10 | Non-fatigue |
| 6034 | Non-fatigued | 27 | 16 | Fatigue |
| 6036 | Non-fatigued | 1 | 14 | Non-fatigue |
| 6042 | Fatigued | 30 | 16 | Fatigue |
| 6045 | Non-fatigued | 15 | 10 | Non-fatigue |
| 6046 | Fatigued | 54 | 20 | Fatigue |
| 6053 | Fatigued | 31 | 16 | Fatigue |
| 6055 | Non-fatigued | 0 | 14 | Non-fatigue |
| 6059 | Fatigued | 42 | 20 | Fatigue |
| 6060 | Non-fatigued | 4 | 12 | Non-fatigue |
| 6063 | Non-fatigued | 27 | 9 | Non-fatigue |
| 6064 | Fatigued | 32 | 9 | Non-fatigue |
| 6065 | Non-fatigued | 13 | 11 | Non-fatigue |
| 6078 | Fatigued | 34 | 8 | Non-fatigue |
| 6079 | Non-fatigued | 0 | 14 | Non-fatigue |
| 6081 | Non-fatigued | 24 | 10 | Non-fatigue |
| 6082 | Non-fatigued | 25 | 15 | Fatigue |
| 6085 | Fatigued | 37 | 18 | Fatigue |
| 6086 | Non-fatigued | 3 | 13 | Non-fatigue |

**Table S1-3.** Participant-level FACIT-FS and MOS-HIV energy/fatigue scores.

*Note. The MOS-HIV energy/fatigue dimension score is the sum of Q18–Q21 (range 4–24). The median split (median = 15) was used as the binary reference standard.*

**Supplementary Table S1-4** *Discriminatory performance of selected FACIT-FS cut-offs against the MOS-HIV energy/fatigue reference standard.*

| **Cut-off** | **Sensitivity** | **Specificity** | **PPV** | **NPV** | **Accuracy** | **Youden Index** |
| --- | --- | --- | --- | --- | --- | --- |
| 20 | 1.000 | 0.765 | 0.818 | 1.000 | 0.886 | 0.765 |
| 25 | 1.000 | 0.824 | 0.857 | 1.000 | 0.914 | 0.824 |
| **30** | 0.889 | 0.882 | 0.889 | 0.882 | 0.886 | 0.771 |
| 35 | 0.722 | 1.000 | 1.000 | 0.773 | 0.857 | 0.722 |

*Note. PPV = positive predictive value; NPV = negative predictive value. The pre-specified study threshold of 30 is shown in bold.*

**Figure S1-1.** Receiver operating characteristic (ROC) curve for the modified FACIT-FS total score in discriminating fatigue status defined by the MOS-HIV energy/fatigue dimension median split.
