# Supplementary material for: Integrated multi-omics reveals plasma biomarkers of fatigue in HIV immunological non-responders
Source: Front Cell Infect Microbiol. 2026 Oct 15;16:1932594. doi: 10.3389/fcimb.2026.1932594 (PMC13716617; doi:10.3389/fcimb.2026.1932594)
Supplement: Supplementary file 2 [file Table2.docx]

Supplementary Material 2

**Figure S2-1：Consort subject flow chart**

Note. INR, immunological non-responder; ART, antiretroviral therapy; VL, viral load; FACIT-FS, Functional Assessment of Chronic Illness Therapy–Fatigue Scale (modified 15-item version, total 0–60, higher scores indicate greater fatigue; score > 30 defined fatigue); MOS-HIV, Medical Outcomes Study HIV Health Survey; UPLC-MS/MS, ultra-performance liquid chromatography–tandem mass spectrometry; PBMC, peripheral blood mononuclear cell. No participant in either group had any AIDS-defining condition.

**Table S2-1. Baseline characteristics of the INR-F and INR-NF groups**

| **Characteristic** | **INR-F (n = 18)** | **INR-NF (n = 17)** | **Statistic / Test** | **P value** |
| --- | --- | --- | --- | --- |
| Age, years, mean ± SD | 42.56 ± 6.53 | 40.47 ± 9.01 | t-test | 0.437 |
| Male sex, n (%) | 13 (72.2) | 10 (58.8) | χ² test | 0.404 |
| Weight, kg, median (Q1, Q3) | 52.00 (50.00, 57.50) | 56.00 (52.00, 66.00) | MWU test | 0.180 |
| ART duration, years, mean ± SD | 5.84 ± 3.90 | 4.61 ± 2.83 | t-test | 0.297 |
| ART regimen class, n (%) |  |  | FFH exact | 0.818 |
| NNRTI-based | 8 (44.4) | 8 (47.1) |  |  |
| PI-based | 4 (22.2) | 2 (11.8) |  |  |
| INSTI-based | 6 (33.3) | 7 (41.2) |  |  |
| EFV-containing regimen, n (%) | 6 (33.3) | 8 (47.1) | χ² test | 0.407 |
| Self-reported good adherence, n (%) | 17 (94.4) | 15 (88.2) | Fisher's exact | 0.603 |
| MPR, %, mean ± SD | 88.44 ± 8.61 | 88.57 ± 5.00 | t-test | 0.957 |
| Time since last dose before sampling, h, mean ± SD | 9.87 ± 3.28 | 11.63 ± 4.22 | t-test | 0.177 |
| Statin use, n (%) | 0 (0.0) | 1 (5.9) | Fisher's exact | 0.486 |
| Corticosteroid use, n (%) | 2 (11.1) | 1 (5.9) | Fisher's exact | 1.000 |
| Traditional Chinese medicine use, n (%) | 0 (0.0) | 3 (17.6) | Fisher's exact | 0.104 |
| Other concomitant medications, n (%) | 2 (11.1) | 1 (5.9) | Fisher's exact | 1.000 |
| Viral Load (copies/ml) | 38.00 (28.00, 44.00) | 35.00 (30.00, 41.00) | Z = 0.28 | 0.796 |

*Note: SD, standard deviation; Q1/Q3, first/third quartile; MWU, Mann–Whitney U; FFH exact, Fisher–Freeman–Halton exact test; MPR, medication possession ratio; ART, antiretroviral therapy; NNRTI, non-nucleoside reverse transcriptase inhibitor; PI, protease inhibitor; INSTI, integrase strand transfer inhibitor; EFV, efavirenz. The chi-square test was applied only when all expected cell counts were ≥ 5; otherwise exact tests were used.*

**Table S2-2. CD4+and CD8+ T-cell counts of the INR-F and INR-NF groups**

| **Characteristic** | **Fatigue group (n=17)** | **Non-fatigue group (n=17)** | **Statistic** | **P value** |
| --- | --- | --- | --- | --- |
| CD4+ T cell count at ART initiation (cells/μl) | 193.6 ± 63.6 | 299.3 ± 131.8 | t = -2.98 | 0.005 |
| Nadir CD4+ T cell count (cells/μl) | 139.2 ± 69.5 | 199.9 ± 67.6 | t = -2.58 | 0.015 |
| CD4+ T cell count at enrollment (cells/μl) | 302.7 ± 57.6 | 311.4 ± 56.6 | t = -0.44 | 0.662 |
| CD4+/CD8+ ratio | 0.35 (0.23, 0.53) | 0.29 (0.20, 0.42) | Z = 0.53 | 0.605 |

Data are presented as mean ± standard deviation or median (P25, P75). a Student’s t-test; b Mann-Whitney U test.

**Figure S2-2. Figure 1. Comparison of baseline CD4+ and CD8+ T cell counts at ART initiation between the fatigue and non-fatigue groups.** Violin plots show the distribution of CD4+ (left) and CD8+ (right) T cell counts (cells/μl) at the initiation of antiretroviral therapy (ART) in the fatigue group (red) and the non-fatigue group (green). Dashed lines indicate the median and quartiles. Comparisons between groups were performed using the Mann–Whitney U test, with P values indicated above each plot.

Figure S2-3. Per-participant CD4+ T-cell counts at ART initiation, nadir, and enrollment in the INR-F and INR-NF groups. Heatmap of individual CD4+ T-cell counts (cells/µL) at three time points; each column represents one participant (case number shown on top), and each row represents a time point. Colour intensity is proportional to the CD4+ T-cell count. INR-F, fatigued immunological non-responders (n = 18, red); INR-NF, non-fatigued immunological non-responders (n = 17, green).
