# Supplementary material for: Integrated multi-omics reveals plasma biomarkers of fatigue in HIV immunological non-responders
Source: Front Cell Infect Microbiol. 2026 Oct 15;16:1932594. doi: 10.3389/fcimb.2026.1932594 (PMC13716617; doi:10.3389/fcimb.2026.1932594)
Supplement: Supplementary file 3 [file Table3.docx]

Supplementary Material 3

**FigureS3-1：Figure S3-1. Plasma metabolomic analysis of fatigued (INR-F) and non-fatigued (INR-NF) HIV immunological non-responders. (A) OPLS-DA score plot showing group separation (orange, fatigued, n = 18; green, non-fatigued, n = 17; ellipses, 95% confidence regions). (B) Permutation test (n = 200) of the OPLS-DA model; R² = (0.0, 0.736) and Q² = (0.0, −0.281) indicate no overfitting. (C) Volcano plot of metabolites; red and blue dots indicate up-regulated (n = 6) and down-regulated (n = 2) metabolites, respectively (VIP > 1, P < 0.05, |log₂FC| > 0.263). (D) Heatmap of the eight differential metabolites, with Z-score-normalised abundance and hierarchical clustering.**

| **ID** | **Ion mode** | **Metabolites** | **KEGG** | **VIP** | **Average(Fatigued)** | **Average(Non-Fatigued)** | **FoldChange** | **log2FoldChange** | **Regulation** | **p-value** | **q-value** |
| --- | --- | --- | --- | --- | --- | --- | --- | --- | --- | --- | --- |
| 549 | pos | DL-arginine | C00792 | 8.774465381 | 6595055.875 | 9093352.213 | 0.725261237 | -0.463427351 | Down | 0.013400279 | 0.784617585 |
| 250 | pos | 4-ketopimelic acid |  | 5.390856936 | 3911152.855 | 1865809.735 | 2.096222773 | 1.067792045 | Up | 0.04006094 | 0.784617585 |
| 856 | pos | N-nervonoyl-d-erythro-sphingosylphosphorylcholine |  | 2.77809748 | 2789921.511 | 2261964.452 | 1.233406435 | 0.302648278 | Up | 0.03109839 | 0.784617585 |
| 441 | pos | Cholesterol | C00187 | 2.312862195 | 608525.6144 | 265378.9478 | 2.29304404 | 1.197264063 | Up | 0.016330601 | 0.784617585 |
| 59 | pos | 1-hexadecyl-sn-glycero-3-phosphocholine |  | 2.294307429 | 748301.384 | 514222.8653 | 1.455208304 | 0.541225681 | Up | 0.023603575 | 0.784617585 |
| 47 | pos | 1-(1z-octadecenyl)-sn-glycero-3-phosphocholine |  | 1.411936345 | 418145.85 | 328066.9706 | 1.274574668 | 0.350015892 | Up | 0.028324438 | 0.784617585 |
| 121 | pos | 1,2-dipalmitoleoyl-sn-glycero-3-phosphocholine |  | 1.313976647 | 120196.1089 | 186047.3636 | 0.646051127 | -0.630279754 | Down | 0.008600377 | 0.784617585 |
| 494 | pos | D-Pipecolinic acid |  | 1.248343175 | 177180.4519 | 105966.5424 | 1.672041456 | 0.741610618 | Up | 0.025993322 | 0.784617585 |

**TableS3-1:Table S3-1. Differential plasma metabolites between fatigued (INR-F) and non-fatigued (INR-NF) HIV immunological non-responders.**

*Note: VIP, variable importance in projection from the OPLS-DA model; Fold change, fatigued/non-fatigued; Regulation, up- or down-regulated in the fatigued group relative to the non-fatigued group; p-value, unadjusted; q-value, Benjamini–Hochberg FDR-adjusted. Metabolites were screened with the criteria VIP > 1, p < 0.05, and |log₂(fold change)| > 0.263.*

**TableS3-2:KEGG pathway enrichment analysis of differential plasma metabolites between fatigued (INR-F) and non-fatigued (INR-NF) HIV immunological non-responders.**

| id | Classification_level1 | Classification_level2 | Term | PopTotal | p-value | q-value | Enrichment_score |
| --- | --- | --- | --- | --- | --- | --- | --- |
| hsa05217 | Human Diseases | Cancer: specific types | Basal cell carcinoma | 3224 | 0.000620347394540866 | 0.00992555831265385 | 1612 |
| hsa04979 | Organismal Systems | Digestive system | Cholesterol metabolism | 3224 | 0.00433838978372714 | 0.0214653415137838 | 230.285714285714 |
| hsa05417 | Human Diseases | Cardiovascular disease | Lipid and atherosclerosis | 3224 | 0.00619481256385412 | 0.0214653415137838 | 161.2 |
| hsa04927 | Organismal Systems | Endocrine system | Cortisol synthesis and secretion | 3224 | 0.00743146537487638 | 0.0214653415137838 | 134.333333333333 |
| hsa04975 | Organismal Systems | Digestive system | Fat digestion and absorption | 3224 | 0.00743146537487638 | 0.0214653415137838 | 134.333333333333 |
| hsa04934 | Human Diseases | Endocrine and metabolic disease | Cushing syndrome | 3224 | 0.00804950306766894 | 0.0214653415137838 | 124 |
| hsa04913 | Organismal Systems | Endocrine system | Ovarian steroidogenesis | 3224 | 0.012370377613139 | 0.0215784935691258 | 80.6 |
| hsa04148 | Cellular Processes | Transport and catabolism | Efferocytosis | 3224 | 0.0129868755047661 | 0.0215784935691258 | 76.7619047619048 |
| hsa04925 | Organismal Systems | Endocrine system | Aldosterone synthesis and secretion | 3224 | 0.0129868755047661 | 0.0215784935691258 | 76.7619047619048 |
| hsa04976 | Organismal Systems | Digestive system | Bile secretion | 3224 | 0.0136031809212477 | 0.0215784935691258 | 73.2727272727273 |
| hsa05200 | Human Diseases | Cancer: overview | Pathways in cancer | 3224 | 0.014835214328774 | 0.0215784935691258 | 67.1666666666667 |
| hsa04977 | Organismal Systems | Digestive system | Vitamin digestion and absorption | 3224 | 0.0228246651509894 | 0.0304328868679858 | 43.5675675675676 |
| hsa00120 | Metabolism | Lipid metabolism | Primary bile acid biosynthesis | 3224 | 0.0289482619109396 | 0.0356286300442333 | 34.2978723404255 |
| hsa00100 | Metabolism | Lipid metabolism | Steroid biosynthesis | 3224 | 0.0350526111563217 | 0.0400601270357963 | 28.280701754386 |
| hsa00470 | Metabolism | Metabolism of other amino acids | D-Amino acid metabolism | 3224 | 0.0423524235315497 | 0.045175918433653 | 23.3623188405797 |
| hsa00140 | Metabolism | Lipid metabolism | Steroid hormone biosynthesis | 3224 | 0.0616830873629289 | 0.0616830873629289 | 15.960396039604 |

*Note: Classification level 1 and level 2 refer to the KEGG pathway hierarchy categories; Pop total, total number of metabolites annotated in the background set; Enrichment score, ratio of enriched metabolites to background metabolites in the pathway. p-values were derived from hypergeometric tests, and q-values were Benjamini–Hochberg FDR-adjusted.*

**Table S3-3.** **Analysis of different metabolites in different groups.**

| **Metabolites Name** | **ID** | **(INR– F) VS Normal** | | | **(INR– NF) VS Normal** | | |
| --- | --- | --- | --- | --- | --- | --- | --- |
|  |  | **log2FoldChange** | **p-value** | **VIP** | **log2FoldChange** | **p-value** | **VIP** |
| 1-oleoyl-2-myristoyl-sn-glycero-3-phosphocholine | 81 | 0.88 | 0.07 | 1.67 | 1.51 | 0.05 | 3.14 |
| 1,2-dipalmitoleoyl-sn-glycero-3-phosphocholine | 121 | -1.36 | 0.00 | 1.59 | -0.73 | 0.01 | 1.10 |
| 1,3,4,6,7,8-hexahydro-1-methyl-2h-pyrimido[1,2-a]pyrimidine | 126 | 1.26 | 0.00 | 1.14 | 1.55 | 0.00192787814834475 | 1.25 |
| 4-[5-[[4-[5-[acetyl(hydroxy)amino]pentylamino]-4-oxobutanoyl]-hydroxyamino]pentylamino]-4-oxobutanoic acid | 235 | -1.28 | 0.00 | 1.90 | -1.12 | 0.00 | 1.88 |
| 4,4'-thiodianiline | 258 | -2.26 | 0.00 | 2.78 | -2.41 | 0.00 | 2.99 |
| 5-aminovaleric acid betaine | 268 | -1.63 | 0.00 | 1.93 | -1.40 | 0.00 | 1.97 |
| 9,10-dihydroxy-12z-octadecenoic acid | 302 | -1.08 | 0.00 | 1.07 | -0.67 | 0.01 | 0.86 |
| Adrenosterone | 318 | -1.85 | 0.00 | 1.78 | -2.09 | 0.00 | 2.00 |
| Artemisinin | 359 | -2.82 | 0.00 | 2.07 | -2.87 | 0.00 | 2.21 |
| Beta-octylglucoside | 397 | -1.21 | 0.00 | 2.03 | -1.30 | 0.00 | 2.41 |
| Cholesterol | 441 | 1.84 | 0.01 | 1.89 | 0.65 | 0.27 | 0.74 |
| D-Pipecolinic acid | 494 | -0.42 | 0.12 | 0.63 | -1.16 | 0.00 | 1.46 |
| Dereplicator identification - rhapsamine | 515 | -1.47 | 0.00 | 1.06 | -1.21 | 0.00 | 0.93 |
| Dibucaine | 519 | -2.36 | 0.00 | 3.55 | -2.41 | 0.00 | 3.83 |
| Ethyl 3-hydroxybutyrate | 581 | -2.75 | 0.00 | 4.98 | -2.75 | 0.00 | 5.29 |
| Isobutyric acid | 694 | -2.56 | 0.00 | 2.38 | -2.64 | 0.00 | 2.56 |
| Lpc 18:2 | 766 | -1.29 | 0.00 | 19.21 | -1.18 | 0.00 | 19.10 |
| Lysine | 774 | -1.18 | 0.00 | 1.05 | -1.48 | 0.00 | 1.31 |
| Methyl (1-(cyclohexylmethyl)-1h-indole-3-carbonyl)-l-valinate | 802 | -1.16 | 0.04 | 1.57 | -1.23 | 0.01 | 1.91 |
| N-(1-amino-3,3-dimethyl-1-oxobutan-2-yl)-1-pentyl-1h-indole-3-carboxamide | 826 | -2.36 | 0.00 | 5.08 | -2.39 | 0.00 | 5.45 |
| Paraxanthine | 924 | -4.80 | 0.00 | 3.56 | -4.38 | 0.00 | 3.80 |
| Phenylacetyl-l-glutamine | 953 | 1.20 | 0.03 | 1.32 | 0.90 | 0.04 | 1.06 |
| Piperine | 964 | -5.51 | 0.00 | 1.85 | -3.87 | 0.01 | 2.04 |
| Pirimicarb | 968 | -2.86 | 0.00 | 2.99 | -2.98 | 0.00 | 3.20 |
| Tegaserod | 1066 | -1.53 | 0.00 | 1.19 | -1.67 | 0.00 | 1.30 |
| Tetraethylene glycol | 1074 | -3.07 | 0.00 | 7.57 | -3.14 | 0.00 | 8.06 |
| Thioetheramide-PC | 1084 | -1.28 | 0.00 | 6.48 | -1.14 | 0.00 | 5.99 |
| Trans-3'-hydroxycotinine | 1097 | 4.09 | 0.02 | 2.04 | 3.43 | 0.02 | 1.76 |
| Triethylene glycol | 1104 | -2.35 | 0.00 | 2.63 | -2.39 | 0.00 | 2.79 |
| Tryptophan betaine | 1116 | -1.22 | 0.01 | 1.80 | -0.85 | 0.10 | 1.41 |
| Tuberostemonine | 1117 | -0.77 | 0.00 | 1.06 | -1.19 | 0.00 | 1.62 |
| (3.beta.)-allopregnanolone sulfate | 1169 | -1.59 | 0.00 | 3.92 | -1.50 | 0.00 | 4.09309567319569 |
| 1-(9z,12z-octadecadienoyl)-2-hydroxy-sn-glycero-3-phosphoethanolamine | 1178 | -1.12 | 0.00 | 1.41 | -0.96 | 0.00 | 1.39 |
| 11-dehydrothromboxane b2 | 1224 | -2.69 | 0.00 | 5.02 | -2.24 | 0.01 | 5.18 |
| 14,17,20,23,26,29-dotriacontahexaenoic acid, (14z,17z,20z,23z,26z,29z)- | 1229 | -1.23 | 0.00 | 1.05 | -1.22 | 0.00 | 1.12 |
| 2-furanpropanoic acid, 3-carboxy-4-methyl-5-propyl- | 1253 | -2.58 | 0.00 | 1.30 | -3.54 | 0.00 | 1.52 |
| 3-dehydroepiandrosterone sulfate | 1279 | -2.11 | 0.00 | 9.95 | -1.65 | 0.00 | 9.93 |
| 5.alpha.-androstan-3.alpha.,17.beta.-diol-o-3-.beta.-glucuronic acid | 1316 | -1.35 | 0.00 | 1.01 | -1.27 | 0.00 | 1.08 |
| 5.alpha.-pregnan-3.alpha.,17-diol-20-one 3-sulfate | 1317 | -1.07 | 0.00 | 1.01 | -1.05 | 0.00 | 1.06 |
| Biocytin | 1368 | -2.49 | 0.00 | 2.21 | -2.32 | 0.00 | 2.29 |
| cis-9-Palmitoleic acid | 1386 | 2.11 | 0.02 | 2.44 | 1.81 | 0.01 | 2.43 |
| Efavirenz | 1434 | 13.24 | 0.01 | 4.56 | 13.60 | 0.01 | 6.63 |
| Pc(16:1e/9-hode) | 1639 | -1.13 | 0.00 | 2.70 | -1.18 | 0.00 | 2.86 |
| Theophylline | 1744 | -3.36 | 0.00 | 1.31 | -3.02 | 0.00 | 1.40 |

**Table S3-4.** **Reference table of down-regulated KEGG pathways in the metabolome compared with different groups.**

| **ID** | **KEGG**  **Term** | **(INR– F) VS Normal** | | **(INR– NF) VS Normal** | |
| --- | --- | --- | --- | --- | --- |
|  |  | p-value | Enrichment  score | p-value | Enrichment  score |
| hsa00310 | Lysine degradation | -0.755580207 | 5.233766234 | -1.220840034 | 7.98019802 |
| hsa00232 | Caffeine metabolism | -2.627840289 | 26.6446281 | -2.151643431 | 36.63636364 |
| hsa04974 | Protein digestion and absorption | -3.987463038 | 30.31974922 | -0.728056099 | 7.196428571 |
| hsa04977 | Vitamin digestion and absorption | -0.922939993 | 7.921375921 | -0.701460361 | 5.84057971 |
| hsa04931 | Endocrine and metabolic disease | -0.810770441 | 15.42583732 | -0.698497394 | 5.101265823 |
| hsa05230 | Cancer: overview | -0.792639451 | 9.76969697 | -2.151643431 | 27.79310345 |
| hsa00140 | Steroid hormone biosynthesis | -1.35207871 | 5.803780378 | -0.728056099 | 7.75 |
| hsa00590 | Arachidonic acid metabolism | -0.621317598 | 3.710011507 | -0.679912694 | 4.333333333 |
| hsa02010 | ABC transporters | -2.489256313 | 9.454545455 | -2.151643431 | 27.79310345 |
| hsa00910 | Energy metabolism | -0.810770441 | 14.65454545 | -0.753013744 | 10.89189189 |
| hsa00220 | Amino acid metabolism | -0.792639451 | 12.743083 | - | - |
| hsa00250 | Amino acid metabolism | -0.792639451 | 10.46753247 | - | - |
| hsa00750 | Metabolism of cofactors and vitamins | -0.792639451 | 10.10658307 | - | - |
| hsa00780 | Biotin metabolism | -2.388592293 | 20.21316614 | - | - |
| hsa00470 | D-Amino acid metabolism | -2.863519819 | 12.743083 | - | - |
| hsa04724 | Nervous system | -0.974080023 | 36.63636364 | - | - |
| hsa04727 | Nervous system | -0.974080023 | 32.56565657 | - | - |
| hsa04964 | Excretory system | -0.810770441 | 17.24064171 | - | - |
| hsa04973 | Digestive system | -0.792639451 | 11.27272727 | - | - |
| hsa00970 | Aminoacyl-tRNA biosynthesis | -1.893189613 | 11.27272727 | - | - |

**Table S3-5. Reference table for up-regulation of KEGG pathways in the metabolic group compared with different groups.**

| **ID** | **Term** | **(INR– F) VS Normal** | | **(INR– NF) VS Normal** | |
| --- | --- | --- | --- | --- | --- |
|  |  | **value** | **Enrichment_score** | **value** | **Enrichment_score** |
| hsa00360 | Phenylalanine metabolism | -1.06 | 10.97 | -1.225854714 | 16.4489795918367 |
| hsa05231 | Choline metabolism in cancer | -1.692220323 | 48.8484848484849 | - | - |
| hsa05217 | Basal cell carcinoma | -2.730243783 | 137.333333333333 | - | - |
| hsa05417 | Lipid and atherosclerosis | -1.733276166 | 53.7333333333333 | - | - |
| hsa04217 | Necroptosis | -1.778696796 | 59.7037037037037 | - | - |
| hsa04979 | Cholesterol metabolism | -1.887167491 | 76.7619047619048 | - | - |
| hsa04975 | Fat digestion and absorption | -1.654768587 | 44.7777777777778 | - | - |
| hsa04934 | Cushing syndrome | -1.620343287 | 41.3333333333333 | - | - |
| hsa04931 | Insulin resistance | -1.457553496 | 28.280701754386 | -1.631222086 | 42.4210526315789 |
| hsa04927 | Cortisol synthesis and secretion | -1.654768587 | 44.7777777777778 | - | - |
| hsa04713 | Circadian entrainment | - | - | -1.953710183 | 89.5555555555556 |
| hsa00061 | Fatty acid biosynthesis | -1.000563541 | 9.59523809523809 | -1.169281593 | 14.3928571428571 |
| hsa04720 | Long-term potentiation | - | - | -2.062450177 | 115.142857142857 |
| hsa04724 | Glutamatergic synapse | - | - | -2.004660462 | 100.75 |
| hsa05016 | Huntington disease | - | - | -2.129194745 | 134.333333333333 |
| hsa05017 | Spinocerebellar ataxia | - | - | -2.062450177 | 115.142857142857 |
| hsa04068 | FoxO signaling pathway | - | - | -2.208173779 | 161.2 |
| hsa05033 | Nicotine addiction | - | - | -2.062450177 | 115.142857142857 |
| hsa05030 | Cocaine addiction | - | - | -2.004660462 | 100.75 |
| hsa04148 | Efferocytosis | -3.224154936 | 51.1746031746032 | - | - |
